# Supplementary material for: DHX9 maintains epithelial homeostasis by restraining R-loop-mediated genomic instability in intestinal stem cells
Source: Nat Commun. 2024 Apr 9;15:3080. doi: 10.1038/s41467-024-47235-2 (PMC11004185; doi:10.1038/s41467-024-47235-2)
Supplement: Supplementary file 1 — Supplementary Information [file 41467_2024_47235_MOESM1_ESM.pdf]

Supplementary information

DHX9 maintains epithelial homeostasis by restraining R-loop-mediated genomic instability in intestinal stem cells

Xingxing Ren[1,2,3], Qiuyuan Liu[4], Peirong Zhou[3], Tingyue Zhou[2], Decai Wang[2], Qiao Mei[4], Richard A. Flavell[5,6], Zhanju Liu[7,*], Mingsong Li[3,*], Wen Pan[1,2,*], Shu Zhu[1,2,8,*]

Table of contents:

Supplementary Methods

Supplementary Figures 1-22

Supplementary Tables 1-3

Supplementary Methods

R-loop Cut&Tag

The R-loop Cut&Tag assays were conducted with slight modifications from previously described methods[1,2], using the Hyperactive Universal CUT&Tag Assay Kit for Illumina Pro (TD904, Vazyme) as the manufacturer's instructions. Briefly, approximately 5×10^5 IECs were washed in 500 µl of wash buffer and then transferred into a 500 µl low-binding tube containing activated concanavalin A–coated magnetic beads for 10 minutes. The bead-bound cells were then incubated overnight at 4°C with either S9.6 (ENH001, Kerafast, 1:100) or normal mouse IgG primary antibodies in 100 µl of antibody buffer. Following a brief wash with dig-wash buffer, the cells were incubated with goat anti-mouse IgG antibody at room temperature for 30 minutes. Post two washes, the samples were treated with 2 µl of pA/G-Tnp Pro in 98 µl dig-300 buffer and incubated at room temperature for 1 hour. Subsequently, the cells were washed thrice and resuspended in 50 µl of tagmentation buffer, and incubated at 37°C for 1 hour to facilitate tagmentation. DNA fragments were then extracted using DNA extract beads and eluted in 20 µl of ddH2O. For the strand displacement reaction, 10 µl of the eluate was combined with 10 U of Bst 2.0 WarmStart DNA polymerase (NEB, M0538) in 1× Q5 polymerase reaction buffer with 1× Q5 high GC enhancer, and 0.5 µl of 10 mM dNTPs (NEB, N0447), and incubated at 65°C for 30 minutes. The reaction was halted by heating at 80°C for 20 minutes. Sequencing libraries were generated by mixing the reaction with indexed P5 and P7 primers and amplified using the Q5 high-fidelity master mix (NEB, M0491). All libraries were sequenced on the NovaSeq 6000 (Illumina) following the manufacturer's instructions. CUT&Tag reads were aligned to the mouse genome (mm10) and analyzed using the online CUT&Tag tool provided by Vazyme and Basepair NGS Data Analysis Platform.

Single cell RNAseq

The single-cell RNA sequencing (scRNA-seq) methodology for IECs was adapted from previous studies[3], incorporating some modifications. Initially, ileum tissues were collected and thoroughly rinsed with cold PBS to remove residual contents. These tissues were longitudinally opened and then cut into small segments, each approximately 2 mm in length. The tissue fragments were incubated in 10 mM EDTA in PBS at 4°C for 30 minutes with gentle shaking to facilitate cell detachment. Afterward, the tissues were vigorously shaken to dislodge the cells, and the resulting supernatant, designated as fraction 1, was collected into a new conical tube. The remaining tissue underwent a second similar incubation and shaking process to collect additional cells. The supernatants from both rounds were combined, and half of this mixture was filtered through a 70 µm cell strainer to isolate crypts. The combined supernatant and crypts were then centrifuged at 300g for 3 minutes to pellet the cells. The pellets were mixed in a 1:1 ratio to ensure an adequate yield of ISCs. This mixture was then dissociated into a single-cell suspension using TrypLE Express (Invitrogen) and subsequently filtered through a 40 µm strainer to remove larger debris prepared for scRNA-seq.

The single-cell RNA-seq libraries and sequencing for our samples were carried out by Berrygenomics Company (Beijing, CN) using the Chromium Single Cell 3′ Reagent Kits v3 (10x Genomics), adhering to the manufacturer's guidelines. The process began with the isolation of single cells and their encapsulation in droplets, followed by barcoding and reverse transcription. Amplified cDNA was then utilized for the construction of 3' gene expression libraries. Specifically, 50 ng of the amplified cDNA underwent fragmentation and end-repair, followed by double-size selection with SPRIselect beads. The libraries were sequenced on a NovaSeq platform (Illumina) to generate 150 bp paired-end reads. Raw sequencing reads were demultiplexed and aligned to the reference genome using the 10X Genomics Cell Ranger pipeline v3 with default settings. For single-cell analyses, we primarily used Cell Ranger and Seurat[4,5], except where

specifically noted. Briefly, unique molecule identifiers were counted for each gene and cell barcode (filtered by CellRanger) to construct digital expression matrices. In the secondary filtration process performed by Seurat, a gene was considered expressed if it was present in more than 3 cells, and each cell was required to express at least 200 genes. Foreign cells were also filtered out during this stage. The Cell Ranger count was employed for alignment, filtering, barcode counting, and UMI counting, generating feature barcode matrices. For secondary gene expression analysis, the Seurat package was utilized for data normalization, dimensionality reduction, clustering, and differential expression analysis. Integrated analysis of datasets was conducted using the Seurat alignment method, canonical correlation analysis (CCA)[6]. For clustering, highly variable genes were identified, and the principal components derived from these genes were used to construct a graph, which was then segmented with a resolution of 0.5.

TUNEL straning

For TUNEL staining, Intestine sections were treated with TUNEL kit (G1501 for FITC detection and G1502 for TRM detection, Servicebio) as per the manufacturer's protocol. Following the staining, TUNEL-positive cells, which exhibit fluorescence, were visualized using a THUNDER imaging system fluorescence microscope (Leica). The intensity and distribution of the TUNEL-positive cells were quantified with Image J software, providing a measure of apoptotic activity within the tissue.

Generation of TetOn-hRNaseH1-Flag HeLa Cell Lines

The generation of TetOn-hRNaseH1-Flag HeLa cell lines was achieved through a lentiviral transduction system. HEK293T cells were initially transfected with the lentiviral vector pLVX-TetOne-hRNaseH1-Flag-IRES-ZsGreen and the packaging plasmids pMD2.G and psPAX2. Forty-eight hours post-transfection, the viral supernatant was collected from the HEK293T cells, followed by filtration through a 0.45 μM filter to remove cell debris. This filtered viral supernatant was then used to infect HeLa cells. During the infection process, polybrene was added at a concentration of 8 μg/ml to enhance viral transduction efficiency. After a suitable incubation period, HeLa cells expressing ZsGreen, indicative of successful transduction, were isolated using flow cytometry-based cell sorting. The resulting TetOn-hRNaseH1-Flag HeLa cell line was designed to conditionally express RNase H1 upon doxycycline (DOX) induction. This system allows for controlled expression of RNase H1, facilitating the study of its role under induced conditions.

Analysis of organoid cell death

The cell death within organoids was assessed using a propidium iodide (PI) staining assay. For this purpose, the organoids were incubated with PI at a concentration of 1 μg/ml for 20 minutes, allowing the PI to stain dead or dying cells. Following the incubation period, images of the stained organoids were captured using a THUNDER Imaging System fluorescence microscope (Leica). To quantify the extent of cell death, the images were analyzed using Image J software. The analysis involved calculating the percentage of the organoid area that was positive for PI staining, which is indicative of cell death.

IF staning of *Lgr5-EGFP-Cre*$^{\mathbf{ERT2}}$ mice

Tissue preparation and fixation: Small intestines or colons were dissected from mice, internally flushed with cold PBS using a gavage needle, and subsequently flushed with 10 ml of cold 1% PFA. The tissues were then incubated in 1% PFA on ice for 1 hour. Each sample was placed on filter paper, longitudinally cut along the mesentery, and carefully unrolled to expose the interior. The unrolled intestines were then gently rolled up, pierced with a needle to retain their shape, and fixed overnight in PFA at 4°C in a centrifuge tube. Sucrose

incubation and OCT embedding: Following fixation, tissues were washed once with 30% sucrose and then transferred to fresh 30% sucrose for overnight incubation at 4°C. The tissues were subsequently removed from the needle and embedded in OCT compound within a rubber plug. To ensure complete OCT infiltration, the samples were left at room temperature for 1 hour, then frozen at -80°C for subsequent sectioning. Staining: Tissue sections were cut, refixation in 4% PFA for 10 minutes, and surrounded by a hydrophobic barrier using a waterproof pen. The sections were washed in PBS, and then blocking buffer (approximately 40 µl for large sections, adjusted as needed) was applied. Incubation occurred at room temperature for 1 hour in a humidified chamber. Primary antibodies were applied for either 3 hours at room temperature or overnight at 4°C in the humidified chamber. This was followed by three PBS washes and the application of secondary antibodies for 1 hour at room temperature. Nuclei were counterstained with DAPI for 5 minutes. After three more PBS washes, mounting medium (20 µl per section, bubble formation avoided) was applied. Samples were then stored in a dark place overnight.

16S rRNA sequencing of fecal microbiota

Fecal samples were collected from mice at the indicated time points using sterile containers. The samples were immediately transferred to the laboratory on ice and stored at -80°C until further processing. DNA extraction from fecal samples was performed using the QIAamp stool DNA mini kit (QIAGEN) following the manufacturer's instructions. The V3-V4 region of the bacterial 16S rRNA gene was amplified using the primer set 341F-806R, which was recommended by the Earth Microbiome Project. The primer sequences included the 515F forward barcode primer: AATGATACGG CGACCACCGA GATCTACACG CTXXXXXXXX XXXXTATGGT AATTGTGTGY CAGCMGCCGC GGTAA and 806R reverse primer: CAAGCAGAAG ACGGCATACG AGATAGTCAG CCAGCCGGAC TACNVGGGTW TCTAAT. The PCR products were purified using the PCR Cleanup kit (QIAGEN) and subsequently subjected to 250 bp paired-end sequencing using the Illumina MiSeq V3 kit. The raw sequencing data underwent bioinformatic analysis for quality control and data processing. Operational taxonomic units (OTUs) were assigned based on a specific sequence similarity threshold using a reference database. Taxonomic classification and diversity analysis were performed using appropriate algorithms and statistical methods.

Supplementary Methods References

1. Wang, K. *et al.* Genomic profiling of native R loops with a DNA-RNA hybrid recognition sensor. *Sci Adv* **7**, doi:10.1126/sciadv.abe3516 (2021).
2. Wang, H., Li, C. & Liang, K. Genome-Wide Native R-Loop Profiling by R-Loop Cleavage Under Targets and Tagmentation (R-Loop CUT&Tag). *Methods Mol Biol* **2528**, 345-357, doi:10.1007/978-1-0716-2477-7_23 (2022).
3. Haber, A. L. *et al.* A single-cell survey of the small intestinal epithelium. *Nature* **551**, 333-339, doi:10.1038/nature24489 (2017).
4. Macosko, E. Z. *et al.* Highly Parallel Genome-wide Expression Profiling of Individual Cells Using Nanoliter Droplets. *Cell* **161**, 1202-1214, doi:10.1016/j.cell.2015.05.002 (2015).
5. Satija, R., Farrell, J. A., Gennert, D., Schier, A. F. & Regev, A. Spatial reconstruction of single-cell gene expression data. *Nat Biotechnol* **33**, 495-502, doi:10.1038/nbt.3192 (2015).
6. Butler, A., Hoffman, P., Smibert, P., Papalexi, E. & Satija, R. Integrating single-cell transcriptomic data across different conditions, technologies, and species. *Nat Biotechnol* **36**, 411-420, doi:10.1038/nbt.4096 (2018).

Supplementary Fig. 1

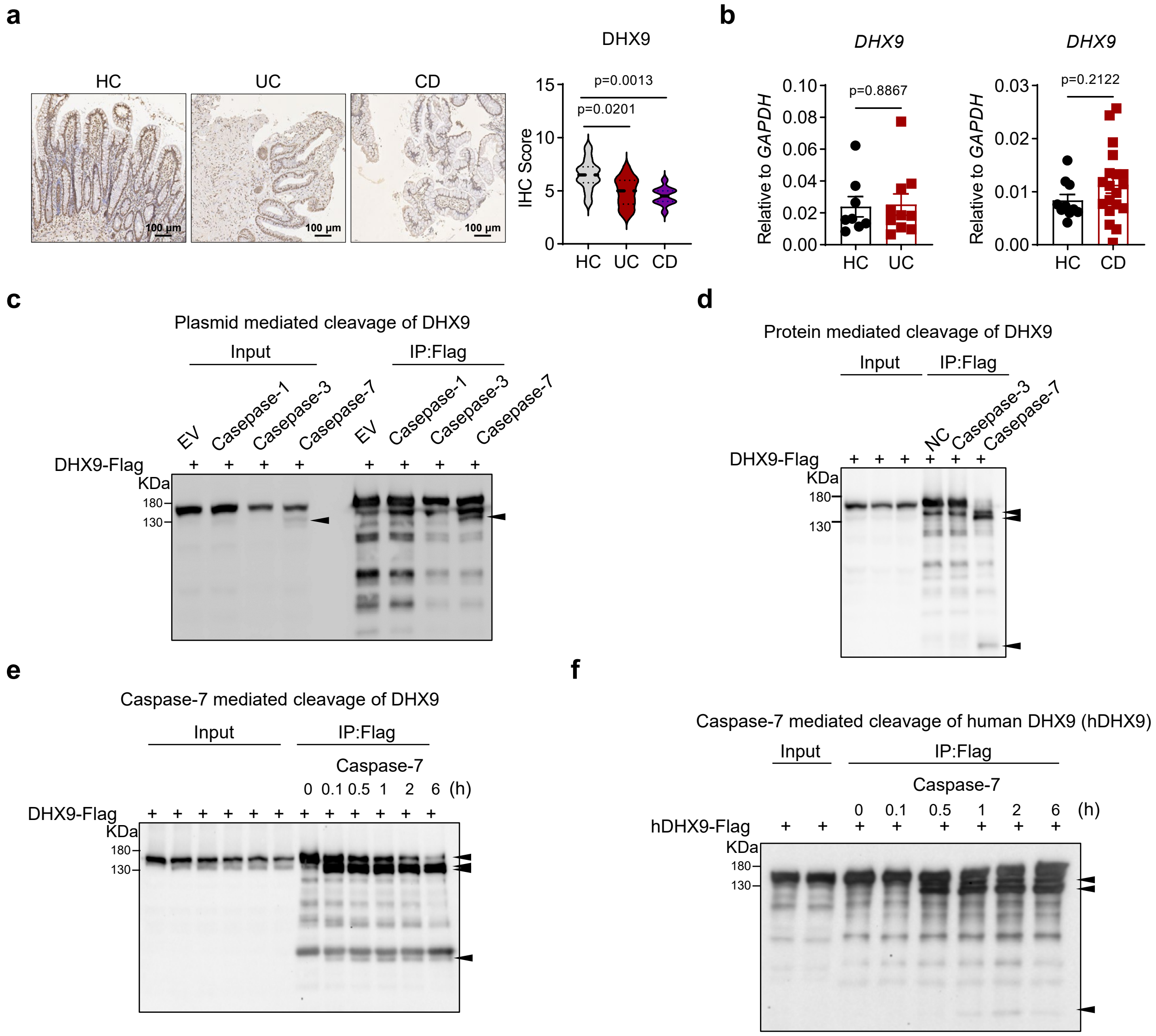

Supplementary Figure 1. Caspase-7-mediated specific cleavage of DHX9 protein. a, Representative images of DHX9 immunohistochemistry (IHC) in human IBD, including ulcerative colitis (UC) and Crohn's disease (CD), as well as healthy control (HC) intestinal samples (n = 10 per group). Quantifications are provided on the right. **b,** Left panel: *DHX9* expression in patients with HC (n = 8) and UC (n = 10) . Right panel: *DHX9* expression in patients with HC (n = 10) and CD (n = 19). **c,** Co-expression of empty vector (EV), Caspase-1, Caspase-3, and Caspase-7 plasmids with Flag-tagged DHX9 in 293T cells. At 24 hours post-transfection, immunoprecipitated with anti-Flag beads and Western blot analysis using anti-Flag antibodies. Black arrows denote the cleavage fragments. **d,** Flag-DHX9 was overexpressed in 293T cells, immunoprecipitated, and incubated with Caspase-3, Caspase-7, or negative control (NC), with subsequent detection by Western blot using anti-Flag antibodies. The black arrows highlight the cleavage fragments. **e,** Time-dependent cleavage of overexpressed Flag-DHX9 by Caspase-7, analyzed via Western blot with anti-Flag antibodies. Black arrows mark the cleaved products. **f,** Time-dependent cleavage of overexpressed human DHX9 (hDHX9) by Caspase-7, analyzed via Western blot with anti-Flag antibodies. Black arrows mark the cleaved products. Data represent mean ± s.e.m. Statistical significance was assessed with a two-tailed unpaired Student's *t*-test. Source data are provided as a Source Data file.

Supplementary Fig. 2

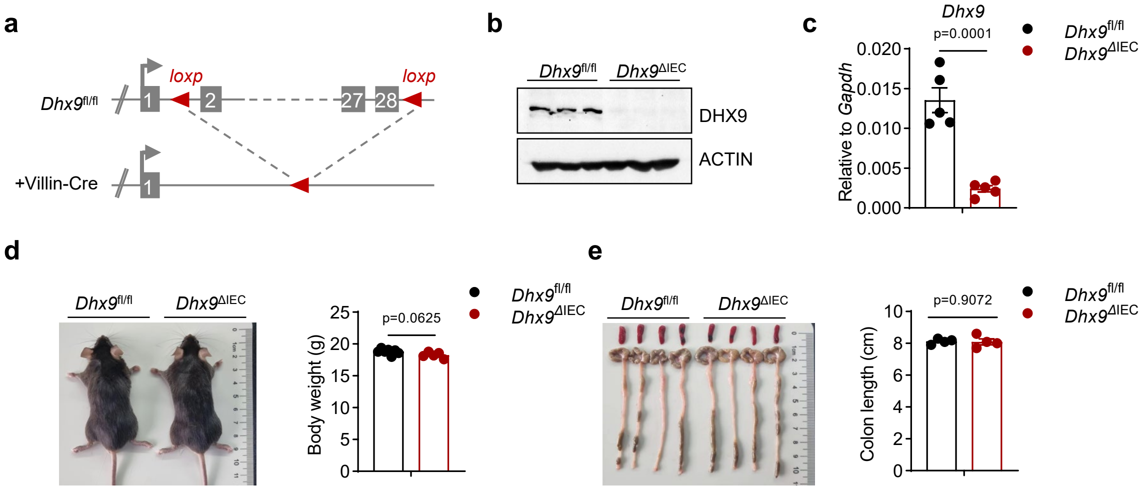

Supplementary Figure 2. Construction of DHX9 conditional knockout mice. **a**, Schematic illustration of the knock-out region of *Dhx9*$^{\Delta IEC}$ mice. **b**, DHX9 deficiency in IECs from *Dhx9*$^{\Delta IEC}$ mice was confirmed by Western blot. **c**, DHX9 deficiency in IECs from *Dhx9*$^{\Delta IEC}$ mice was confirmed by RT-qPCR right (n = 5 per group). **d**, Body weight of 8-week-old *Dhx9*$^{\Delta IEC}$ mice (n = 5) compared with *Dhx9*$^{fl//fl}$ littermates (n = 7). Quantifications are provided on the right. **e**, Measurement of colon length of 8-week-old *Dhx9*$^{\Delta IEC}$ mice compared with *Dhx9*$^{fl//fl}$ littermates, with quantifications provided on the right (n = 4 per group). All data are presented as mean ± s.e.m. Statistical analysis were performed using a two-tailed unpaired Student's *t* test. Source data are provided as a Source Data file.

Supplementary Fig. 3

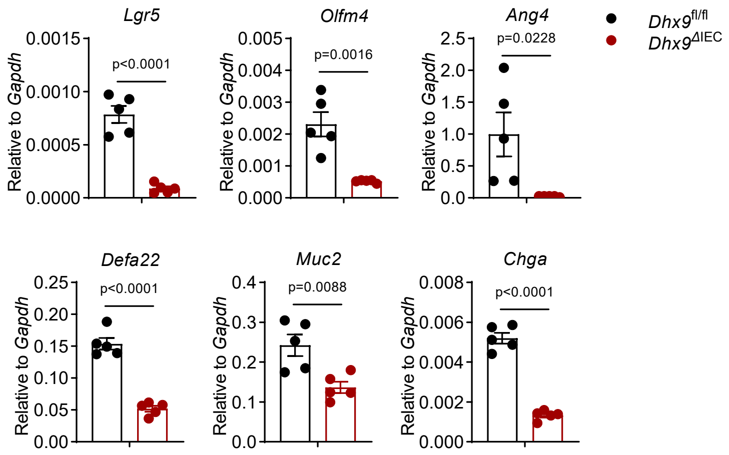

Supplementary Figure 3. Altered Expression of ISCs and secretory cell markers due to DHX9 Deficiency. RT-qPCR analysis of marker gene expression for epithelial cells in IECs from *Dhx9*$^{fl//fl}$ (n = 5) and *Dhx9*$^{\Delta IEC}$ (n = 5) mice at 8 weeks of age. Data represent mean ± s.e.m. Statistical analysis was performed using two-tailed unpaired Student's *t* test. Source data are provided as a Source Data file.

Supplementary Fig. 4

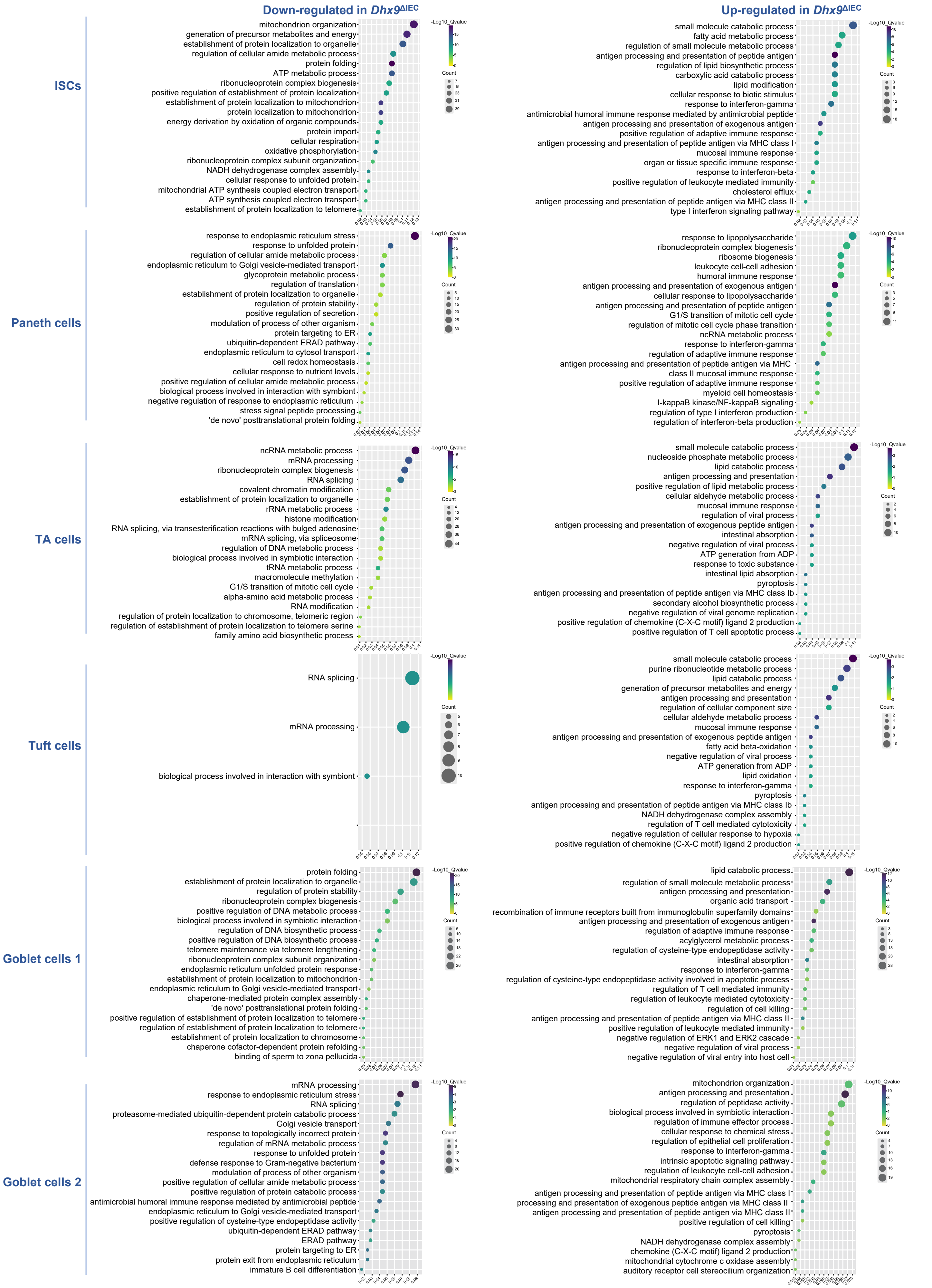

Supplementary Figure 4. Gene Ontology analysis of downregulated and upregulated genes in IEC Subsets of *Dhx9*$^{\Delta IEC}$ mice. Gene ontology analysis of genes showing downregulated and upregulated in ISCs, Paneth cells, TA cells, tuft cells, and goblet cells in *Dhx9*$^{\Delta IEC}$ mice relative to *Dhx9*$^{fl//fl}$ controls. The analysis is based on single-cell RNA sequencing (scRNA-seq) data with statistical significance set at $P < 0.05$, determined by right-tailed Fisher's exact test.

Supplementary Fig. 5

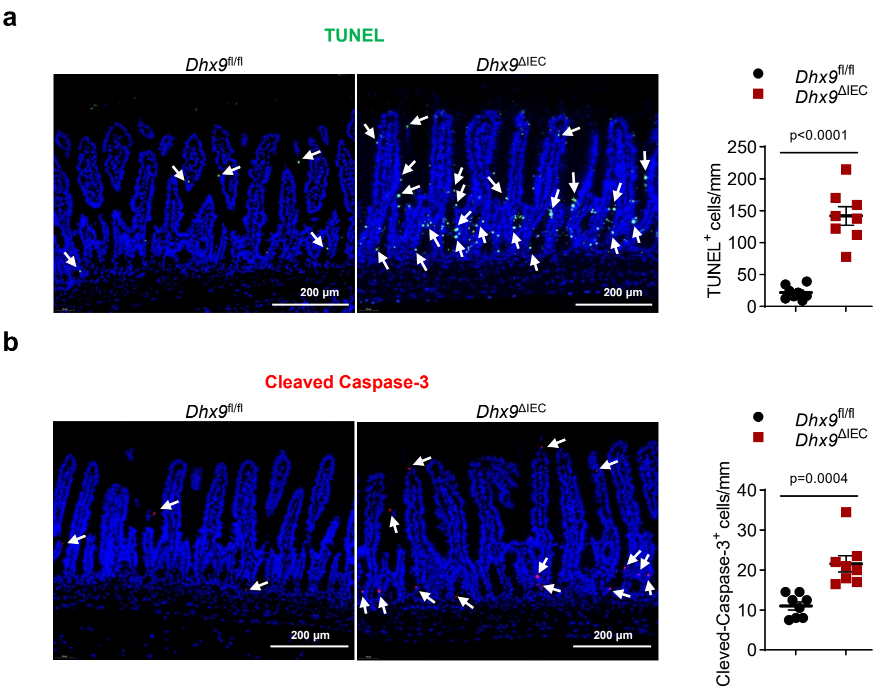

Supplementary Figure 5. Apoptotic markers in the intestine of *Dhx9*$^{\Delta IEC}$ mice. a, TUNEL assay staining of ileum sections from *Dhx9*$^{fl//fl}$ and *Dhx9*$^{\Delta IEC}$ mice at 6 weeks of age to detect DNA fragmentation indicative of apoptosis. Representative images are shown with quantification on the right. n = 8 per genotype. TUNEL-positive cells are indicated with arrows. **b**, IF staining for cleaved caspase-3 on ileum sections from *Dhx9*$^{fl//fl}$ and *Dhx9*$^{\Delta IEC}$ mice at 6 weeks of age. Representative images are shown with quantification on the right. n = 8 per genotype. Cells positive for cleaved caspase-3 are marked with arrows. Data represent mean ± s.e.m. Statistical analysis was performed using two-tailed unpaired Student's *t* test. Source data are provided as a Source Data file.

Supplementary Fig. 6

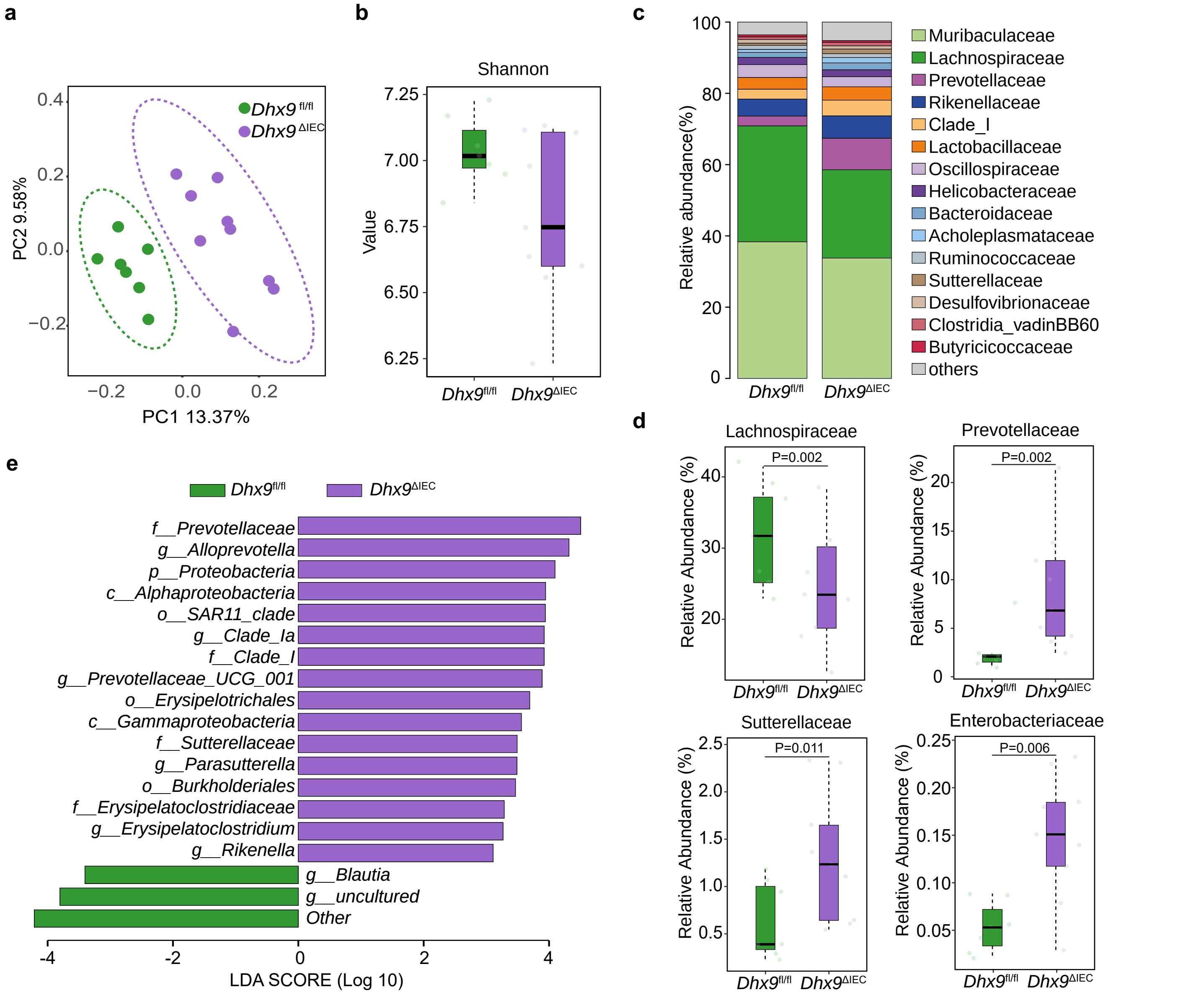

Supplementary Figure 6. The gut microbiota is altered in *Dhx9*$^{\Delta IEC}$ mice. **a**, Fecal 16S rRNA sequencing results for *Dhx9*$^{\Delta IEC}$ mice and their *Dhx9*$^{fl//fl}$ littermates. PCoA of 16s sequencing data. **b**, Alpha diversity. **c**, Relative abundance data for bacterial composition (family-level taxonomy) are presented as a percentage of the total detected sequences. **d**, Box plots of the relative abundance of *Prevotellaceae*, *Sutterellaceae*, *Enterobacteriaceae,* and *Lachnospiraceae,* e, LEfSe analysis to identify differences of bacterial composition in abundant taxa between *Dhx9*$^{\Delta IEC}$ mice and *Dhx9*$^{fl//fl}$ littermates.

Supplementary Fig. 7

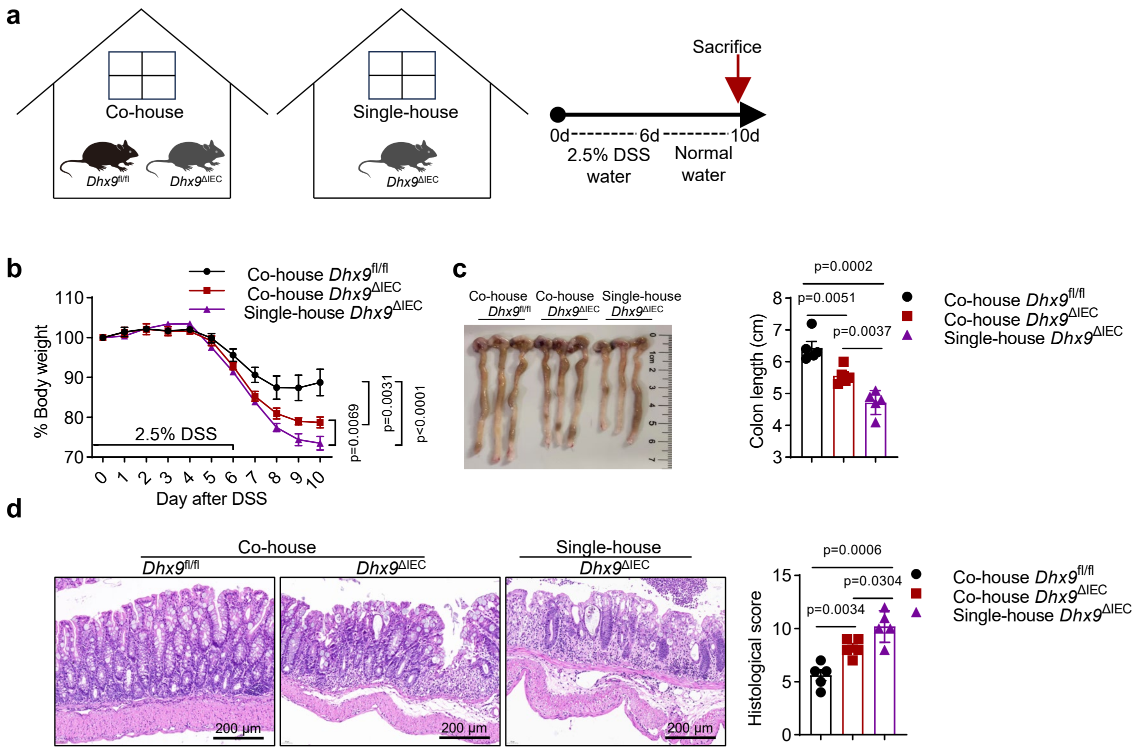

Supplementary Figure 7. Co-house treatment partially alleviates colitis in *Dhx9*$^{\Delta IEC}$ mice. a, Experimental design depicting the induction of colitis using DSS. Co-house *Dhx9*$^{fl//fl}$ (n = 5), Co-house *Dhx9*$^{\Delta IEC}$ (n = 5), and Single-house *Dhx9*$^{\Delta IEC}$ (n = 5) mice were treated with 2.5% DSS for 6 days. **b**, Monitoring of body weight changes throughout the experimental period. **c**, Measurement of colon length upon sacrifice on day 10, with quantifications provided on the right. **d**. Representative H&E staining of colon sections and corresponding histological scores (right). All data are presented as mean ± s.e.m. Statistical analysis were performed using two-way ANOVA analysis with Tukey's multiple comparisons (**b**) and two-tailed unpaired Student's *t* test (**c** and **d**). Source data are provided as a Source Data file.

Supplementary Fig. 8

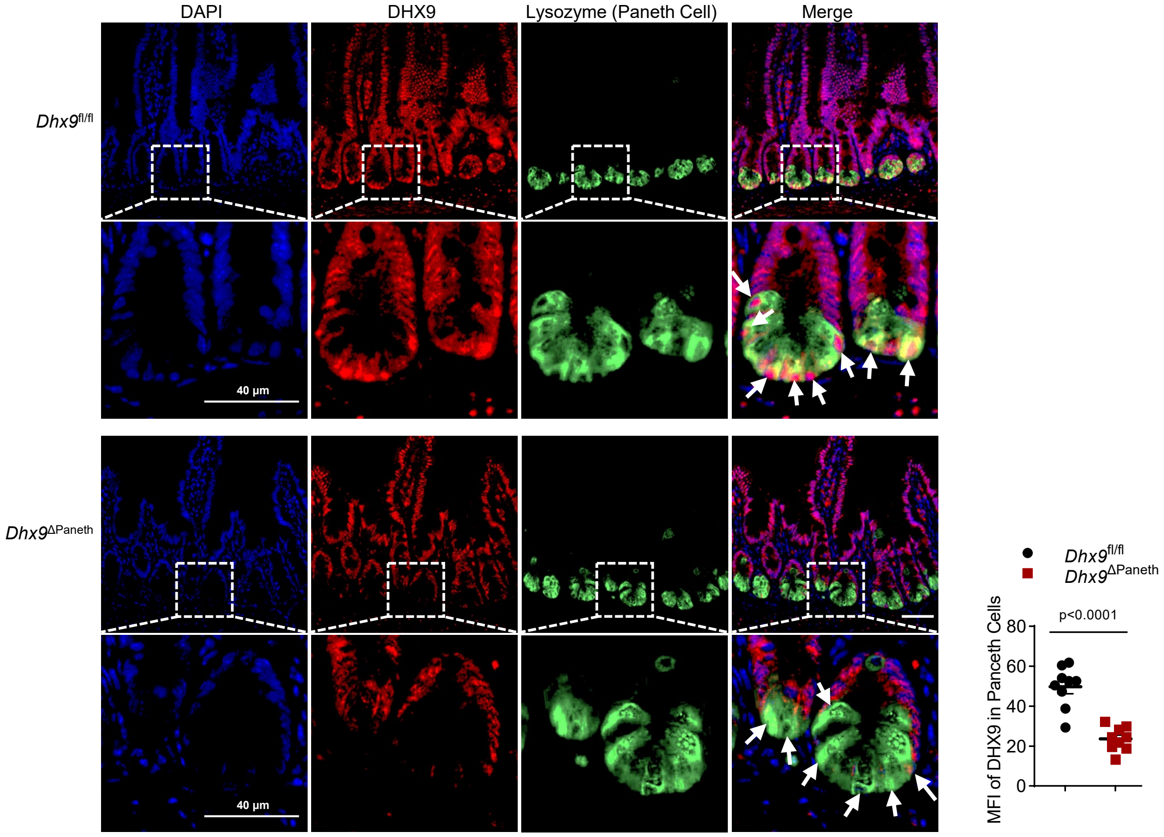

Supplementary Figure 8. Specific knockdown of DHX9 in Paneth cells of the intestines in *Dhx9*$^{\Delta Paneth}$ mice. Representative immunostaining of Lysozyme (green) and DHX9 (red) in ileum sections from *Dhx9*$^{fl//fl}$ and *Dhx9*$^{\Delta Paneth}$ mice at 8 weeks of age. Nuclei are stained with DAPI (blue). Experiment was performed on n = 3 mice individually, with similar results. Mean Fluorescence Intensity (MFI). Scale bars represent 40 µm. Quantifications are provided on the right. Data represent mean ± s.e.m. Statistical analysis were performed using two-tailed unpaired Student's *t* test. Source data are provided as a Source Data file.

Supplementary Fig. 9

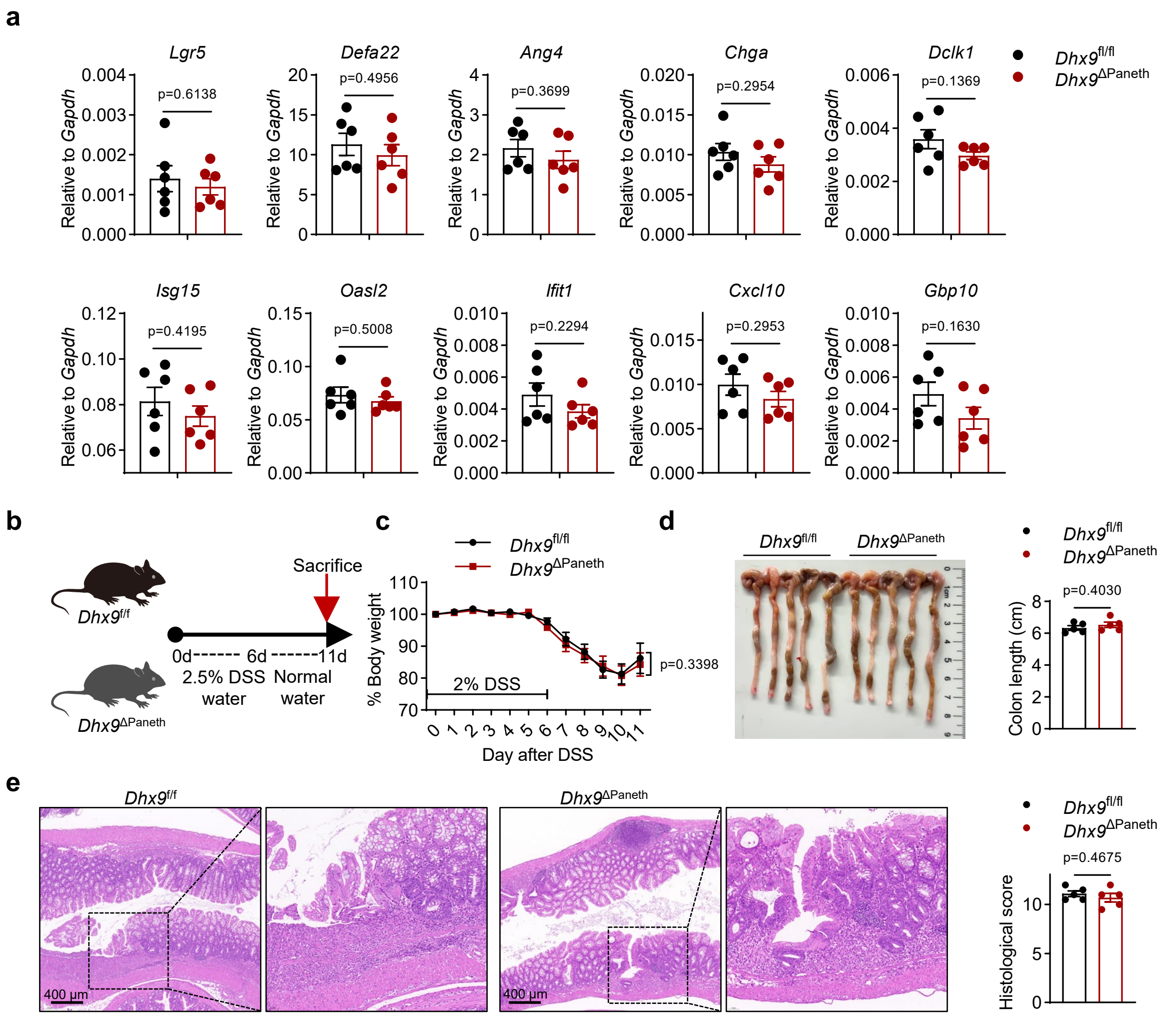

Supplementary Figure 9. Paneth-cell-specific DHX9 signaling is dispensable for DSS-induced colitis. a, RT-qPCR analysis of marker gene expression for epithelial cells and interferon stimulating genes in IECs from *Dhx9*$^{\Delta Paneth}$ mice (n = 6) and their *Dhx9*$^{fl//fl}$ littermates (n = 6) at 8 weeks of age. **b**, Experimental design depicting the induction of colitis using DSS. *Dhx9*$^{fl//fl}$ and *Dhx9*$^{\Delta Paneth}$ mice were treated with 2.5% DSS for 6 days (n = 5 per group). **c**, Monitoring of body weight changes throughout the experimental period. **d**, Measurement of colon length upon sacrifice on day 11, with quantifications provided on the right. **e**. Representative H&E staining of colon sections and corresponding histological scores (right). Scale bar, 400 μm. Statistical analysis were performed using two-way ANOVA analysis with Tukey's multiple comparisons (**c**) and two-tailed unpaired Student's *t* test (**a,d,** and **e**). Source data are provided as a Source Data file.

Supplementary Fig. 10

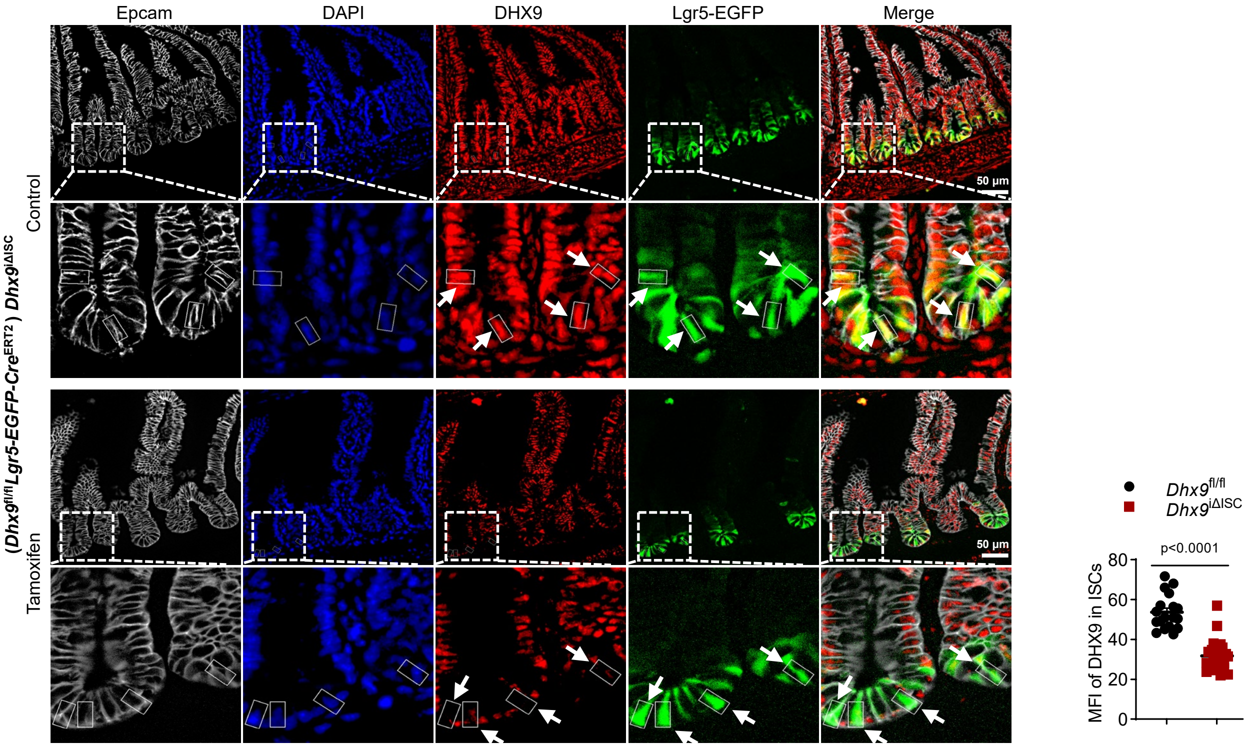

Supplementary Figure 10. Specific knockdown of DHX9 in ISCs of the intestines in *Dhx9*$^{\Delta iISC}$ mice. Representative immunostaining of Epcam (white), DHX9 (red) in ileum sections from *Dhx9*$^{fl//fl}$ and *Dhx9*$^{\Delta iISC}$ mice, which were treated with tamoxifen for 6 days at 8 weeks of age to induce specific gene knockdown. EGFP is derived from endogenous fluorescence. Nuclei are stained with DAPI (blue). Scale bars represent 50 µm. At least of 18 ISCs were counted. Experiment was performed on n = 3 mice individually, with similar results. Quantifications are provided on the right. Data represent mean ± s.e.m. Statistical analysis were performed using two-tailed unpaired Student's *t* test. Source data are provided as a Source Data file.

Supplementary Fig. 11

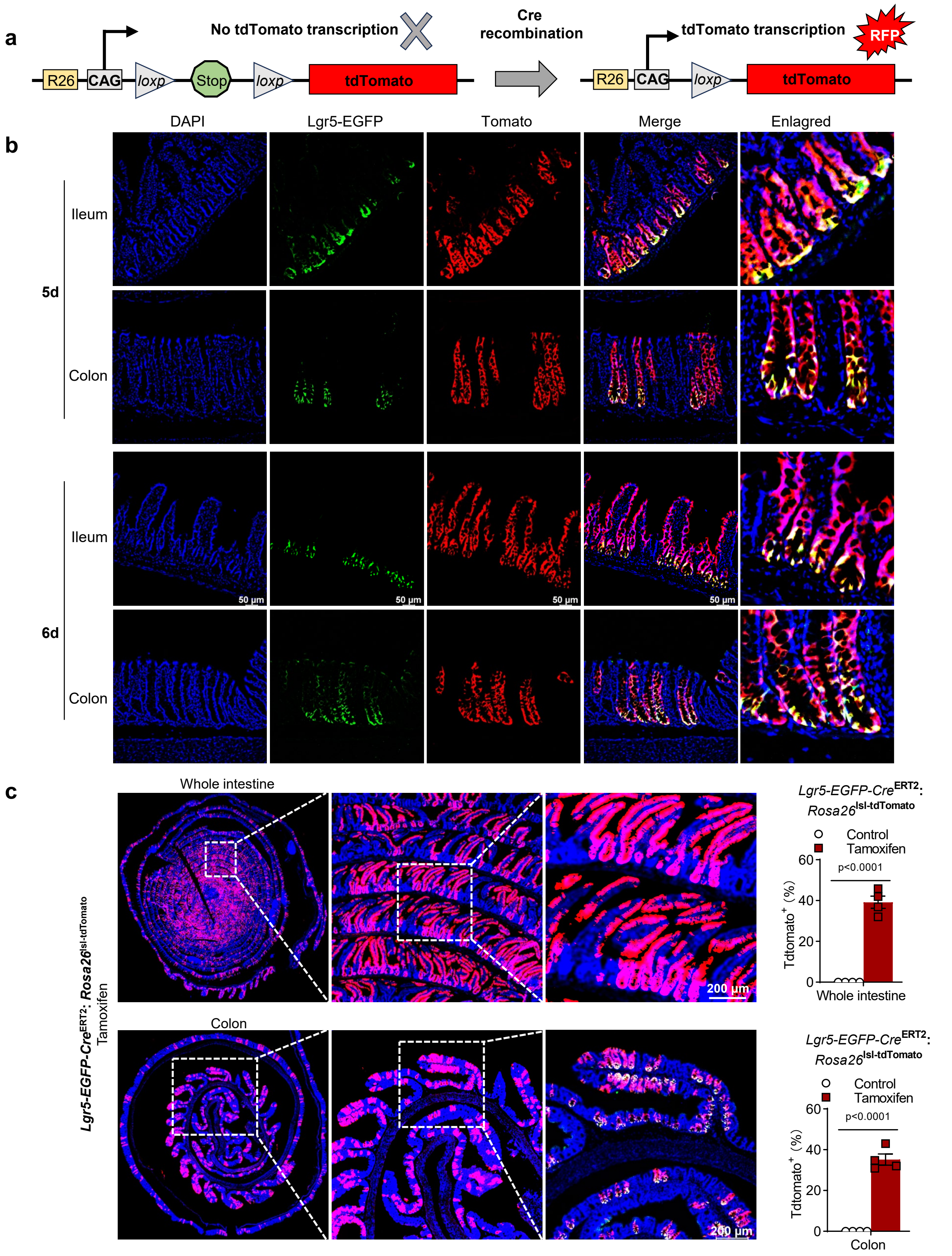

Supplementary Figure 11. The knockout efficiency of *Lgr5-EGFP-Cre*^{ERT2} was tracked by tdTomato fluorescence. a, Diagram of the *loxP*-flanked STOP cassette upstream of tdTomato with and without Cre recombination in *Rosa26*$^{lsl\text{-}tdTomato}$ mice. **b**, Representative image of EGFP and tdTomato in ileum and colon sections from *Lgr5-EGFP-Cre*^{ERT2}: Rosa26$^{lsl\text{-}tdTomato}$ mice, which were treated with tamoxifen for 5 and 6 days at 8 weeks of age. EGFP and tdTomato are derived from endogenous fluorescence. Nuclei are stained with DAPI (blue). Scale bars represent 50 μm. **c**, Representative image of whole intestine or colon from *Lgr5-EGFP-Cre*^{ERT2}: Rosa26$^{lsl\text{-}tdTomato}$ mice, which were treated with tamoxifen for 6 days at 8 weeks of age. tdTomato are derived from endogenous fluorescence. Nuclei are stained with DAPI (blue). n = 4 per group. Data represent mean ± s.e.m. Statistical analysis was performed using two-tailed unpaired Student's *t* test. Source data are provided as a Source Data file.

Supplementary Fig. 12

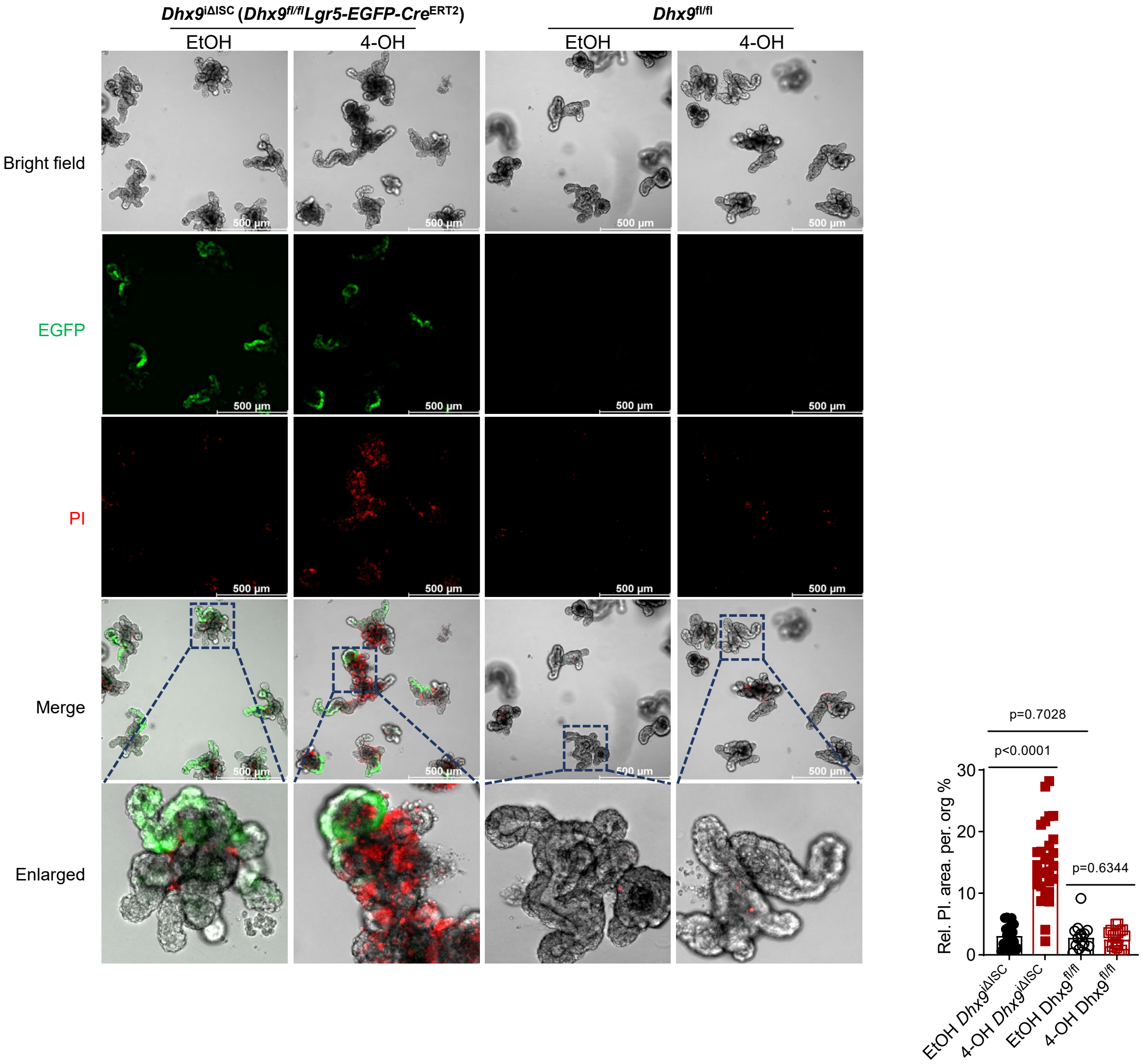

Supplementary Figure 12. 4-OH induced DHX9 knockout increased the mortality of *Dhx9*$^{i\Delta ISC}$ organoids, but did not affect the growth of *Dhx9*$^{fl//fl}$ organoids. Representative images of *Dhx9*$^{i\Delta ISC}$ and *Dhx9*$^{fl//fl}$ ileum organoids, cultured for 5 days. Organoids were treated with EtOH as a control or with 4-OH (200 nM) to induce DHX9 knockout for 24 hours. Propidium iodide (PI) staining traces cell mortality. The right panel provides statistical analysis of the relative PI area per organoid, indicating cell death. At least 15 organoids were counted. Three individual experiments were performed, with similar results. EGFP is derived from endogenous fluorescence. Scale bars represent 500 μm. All data are presented as mean ± s.e.m. Statistical significance was assessed with a two-tailed unpaired Student's *t*-test. Source data are provided as a Source Data file.

Supplementary Fig. 13

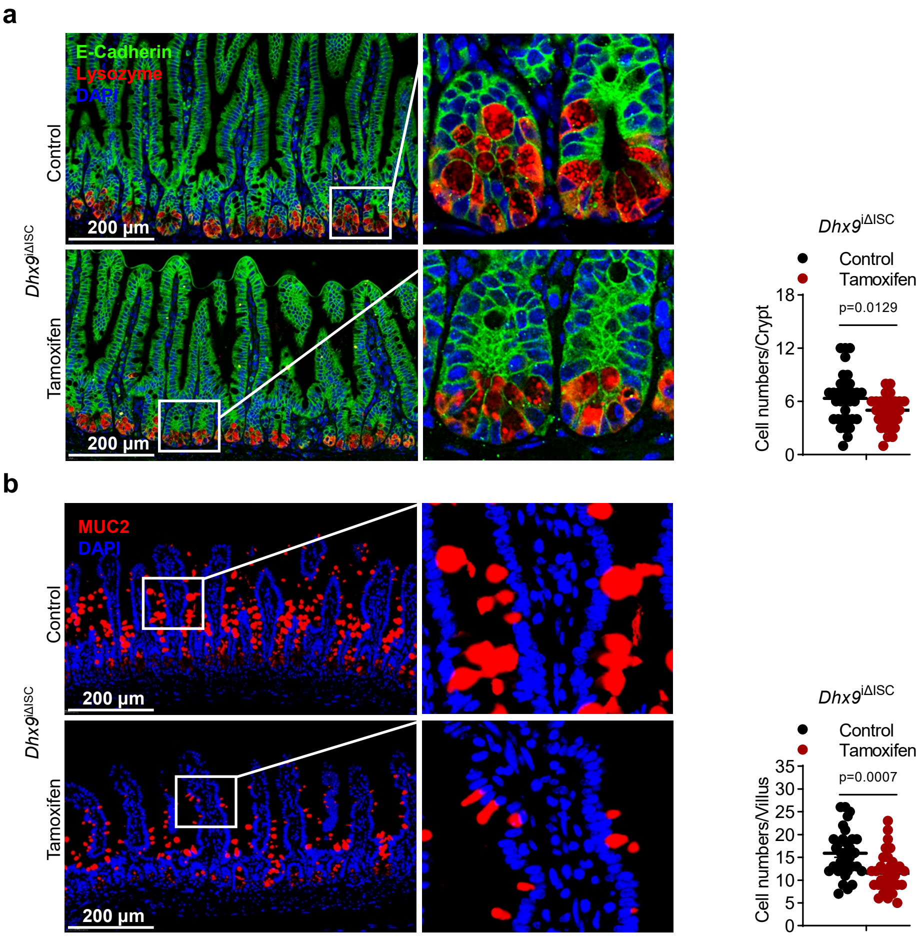

Supplementary Figure 13. Disruption of DHX9 in ISCs induces altered secretory cell lineages. a, Representative immunostaining of Lysozyme (red) and E-cadherin (green) in ileum sections from corn oil-injected (control) and tamoxifen-treated *Dhx9*$^{i\Delta ISC}$ mice at 8 weeks of age. Nuclei are stained with DAPI (blue). Scale bars represent 200 µm. At least 30 crypt-villus axes were counted. Experiment was performed on n = 3 mice individually, with similar results. Quantifications are provided on the right. **b**, Representative immunostaining of MUC2 (red) in ileum sections from corn oil-injected (control) and tamoxifen-treated *Dhx9*$^{i\Delta ISC}$ mice at 8 weeks of age. Nuclei are stained with DAPI (blue). Scale bars represent 200 µm. At least 30 crypt-villus axes were counted. Experiment was performed on n = 3 mice individually, with similar results. Quantifications are provided on the right. All data are presented as mean ± s.e.m. Statistical analysis was performed using a two-tailed unpaired Student's *t* test. Source data are provided as a Source Data file.

Supplementary Fig. 14

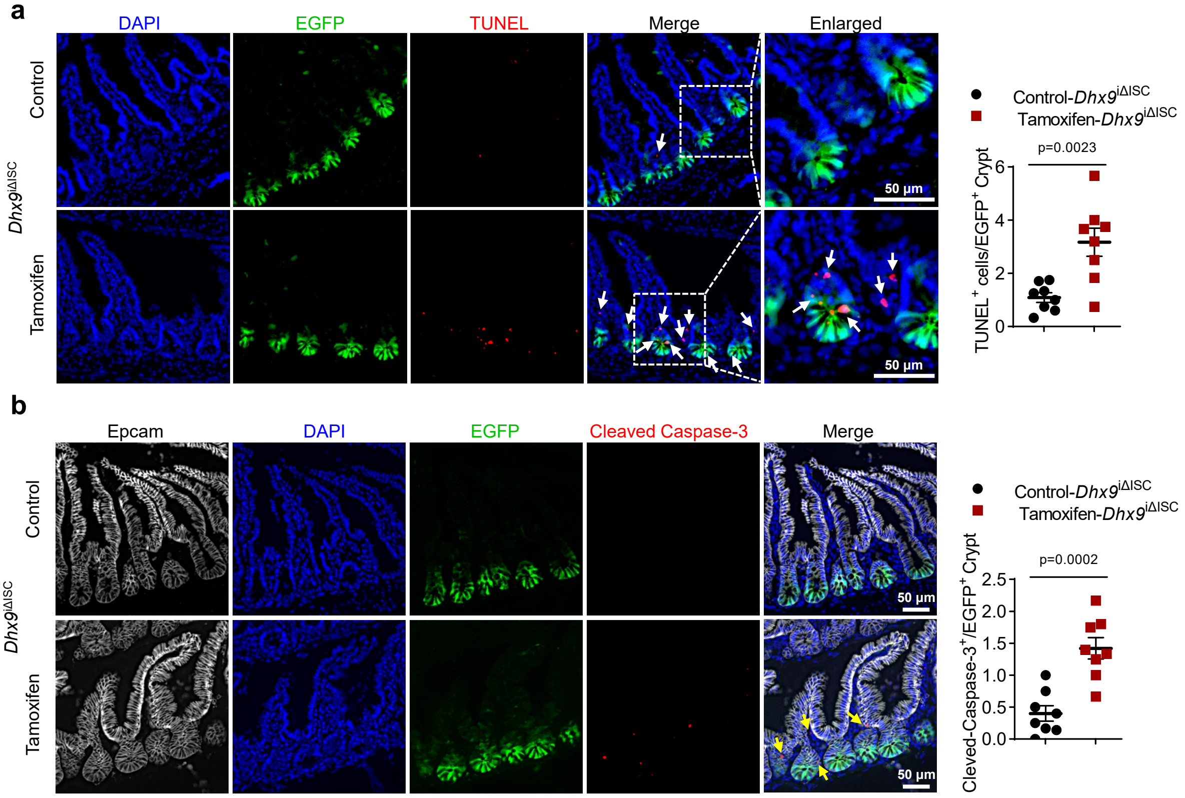

Supplementary Figure 14. Apoptotic markers in the intestine of *Dhx9*$^{i\Delta ISC}$ mice. a, TUNEL assay staining of ileum sections from corn oil-injected (control) and tamoxifen-treated Dhx9$^{i\Delta ISC}$ mice at 8 weeks of age to detect DNA fragmentation indicative of apoptosis. Representative images are shown with quantification on the right. n = 8 per genotype. TUNEL-positive cells are indicated with arrows. Scale bars represent 50 μm. **b**, IF staining for cleaved caspase-3 on ileum sections from corn oil-injected (control) and tamoxifen-treated Dhx9$^{i\Delta ISC}$ mice at 8 weeks of age. Representative images are shown with quantification on the right. n = 8 per genotype. Cells positive for cleaved caspase-3 are marked with arrows. Scale bars represent 50 μm. All data are presented as mean ± s.e.m. Statistical analysis was performed using a two-tailed unpaired Student's *t* test. Source data are provided as a Source Data file.

Supplementary Fig. 15

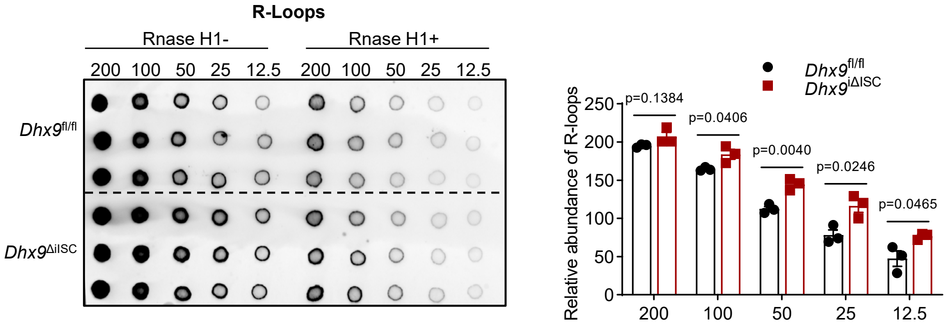

Supplementary Figure 15. R-Loop quantification in ileum crypts following DHX9 knockdown. Dot blot analysis conducted to quantify R-loops in ileum crypts of *Dhx9*$^{i\Delta ISC}$ and *Dhx9*$^{fl//fl}$ mice (n = 3 per group). These mice were treated with tamoxifen for 6 days at 8 weeks of age to induce specific DHX9 knockdown. Consistent amounts of DNA from each sample were applied onto a nitrocellulose membrane. R-loops were detected using the S9.6 antibody, with RNase H1 treatment serving as a negative control. Quantitative results are presented on the right. All data are presented as mean ± s.e.m. Statistical analysis was performed using a two-tailed unpaired Student's *t* test. Source data are provided as a Source Data file.

Supplementary Fig. 16

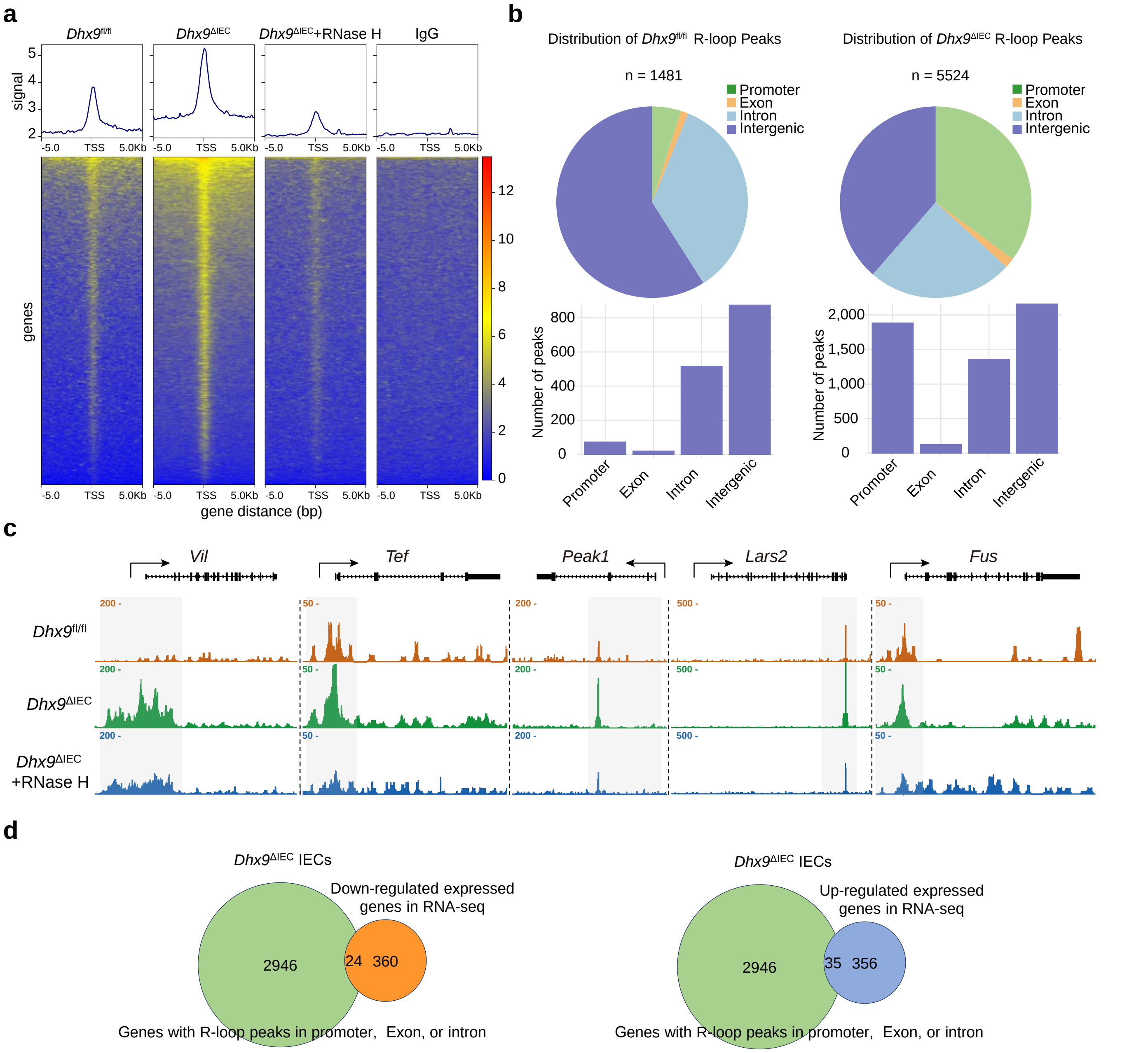

Supplementary Figure 16. Genomic distribution of R-loop peaks in *Dhx9*$^{\Delta IEC}$ IECs. a, Heatmaps depicting the CUT&Tag-seq signal distribution around the transcription start sites (TSS) within a 5 kb range, both upstream and downstream. Signals are shown for S9.6 antibody in *Dhx9*$^{fl//fl}$ IECs, S9.6 antibody in *Dhx9*$^{\Delta IEC}$ IECs, S9.6 antibody in *Dhx9*$^{\Delta IEC}$ IECs that treated with RNase H, and IgG control in *Dhx9*$^{fl//fl}$ IECs. **b**, Genomic distribution variation of R-loop peaks in *Dhx9*$^{fl//fl}$ and *Dhx9*$^{\Delta IEC}$ IECs, as revealed by CUT&Tag-Seq data. The annotations highlight the distribution of these peaks across different genomic regions. **c**, UCSC genome browser tracks of CUT&Tag signals at the *Vil1*, *Tef*, *Peak1*, *Lars2*, and *Fus* loci. **d**, Venn diagrams showing the overlap of R-loop accumulated genes and transcriptionally upregulated or downregulated genes in RNA-seq analysis of DHX9-deficient IECs.

Supplementary Fig. 17

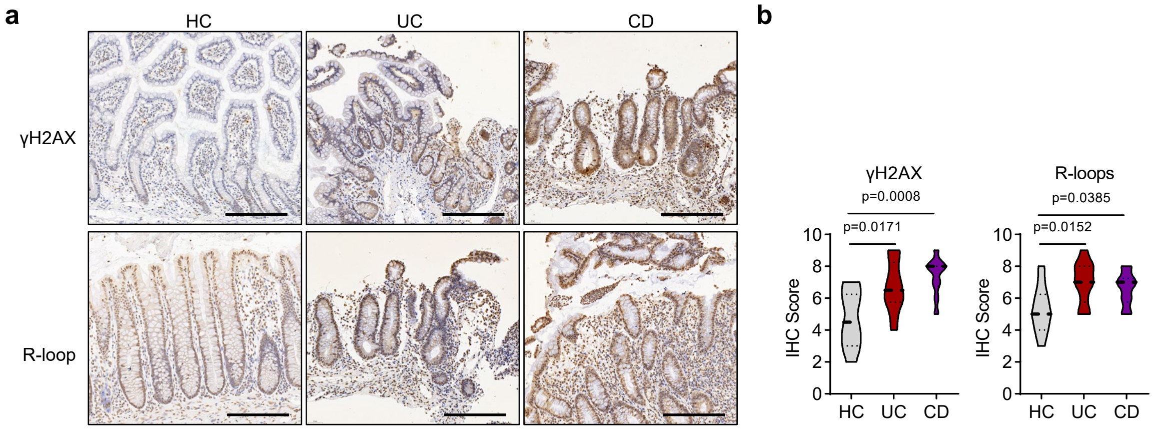

Supplementary Figure 17. IHC of γH2AX and R-loop human IBD samples. a, Representative images of γH2AX and R-loop IHC in human IBD (UC and CD) and healthy control (HC) intestinal samples (n = 10 per group). Scale bars represent 200 μm. **b**, Quantifications of γH2AX and R-loop signals in human IBD and healthy control samples. All data are presented as mean ± s.e.m. Statistical analysis was performed using a two-tailed unpaired Student's *t* test. Source data are provided as a Source Data file.

Supplementary Fig. 18

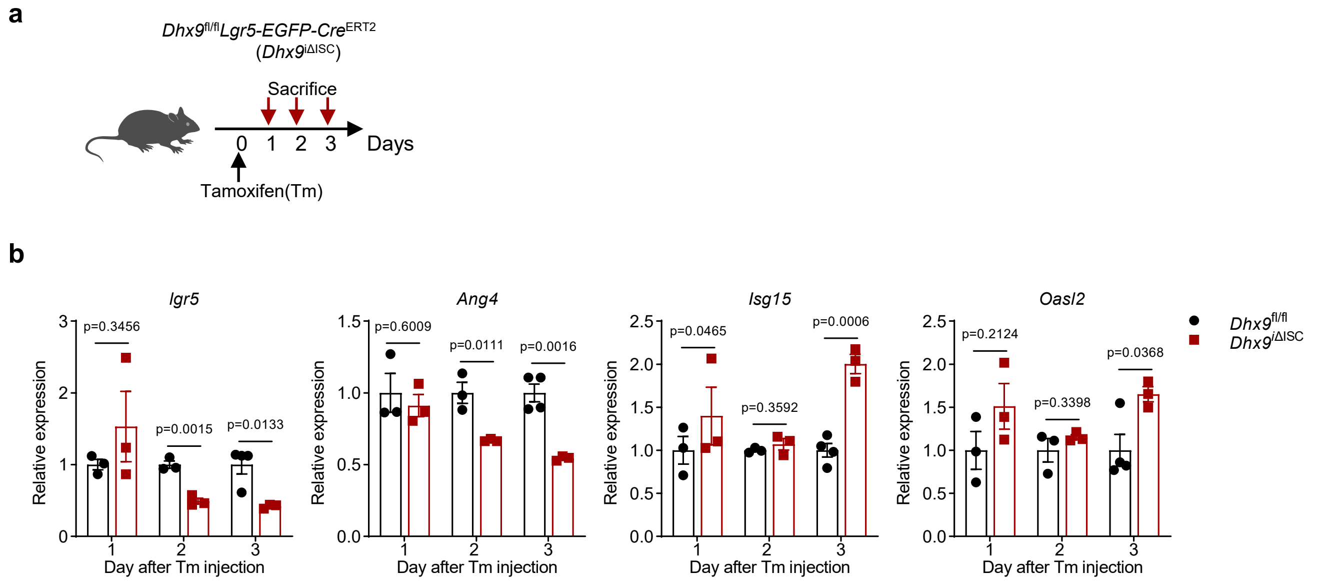

Supplementary Figure 18. Time-course study post-tamoxifen treatment in *Dhx9*$^{\mathrm{i\Delta ISC}}$ mice. a, Schematic diagram illustrating the strategy of tamoxifen (Tm) administration. *Dhx9*$^{\mathrm{i\Delta ISC}}$ mice were injected with tamoxifen and subsequently sacrificed at 1, 2, and 3 days post-injection. **b**, RT-qPCR analysis the expression of *Lgr5*, *Ang4*, *Isg15*, and *Oasl2* in IECs from *Dhx9*$^{\mathrm{i\Delta ISC}}$ mice (n = 10) and their *Dhx9*$^{\mathrm{fl//fl}}$ littermates (n = 9) at 8 weeks of age. All data are presented as mean ± s.e.m. Statistical analysis was performed using a two-tailed unpaired Student's *t* test. Source data are provided as a Source Data file.

Supplementary Fig. 19

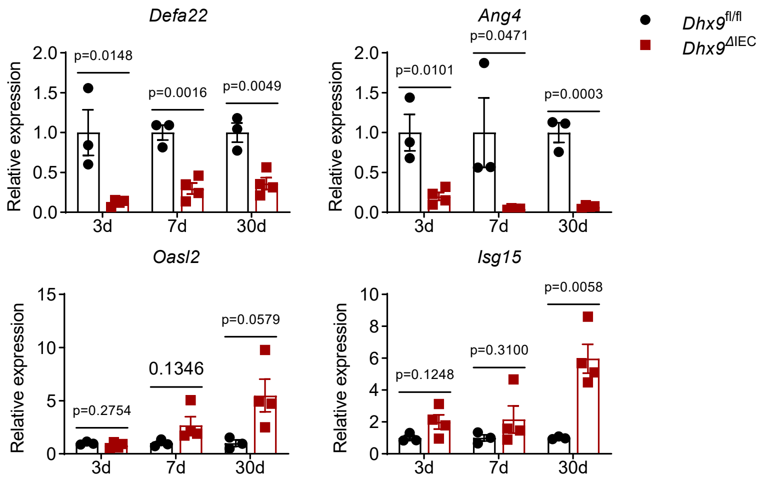

Supplementary Figure 19. Age-related changes in IECs of *Dhx9*$^{\Delta IEC}$ mice. RT-qPCR analysis assessing the expression of *Defa22*, *Ang4*, *Oasl2*, and *Isg15* in IECs from *Dhx9*$^{\Delta IEC}$ mice at different ages. The study included analysis of IECs at 3 days, 7 days, and 30 days after birth. Mice in the experimental group (*Dhx9*$^{\Delta IEC}$) had n = 3 for each time point, while their *Dhx9*$^{fl//fl}$ littermates (control group) had n = 4 for each age group. All data are presented as mean ± s.e.m. Statistical analysis was performed using a two-tailed unpaired Student's *t* test. Source data are provided as a Source Data file.

Supplementary Fig. 20

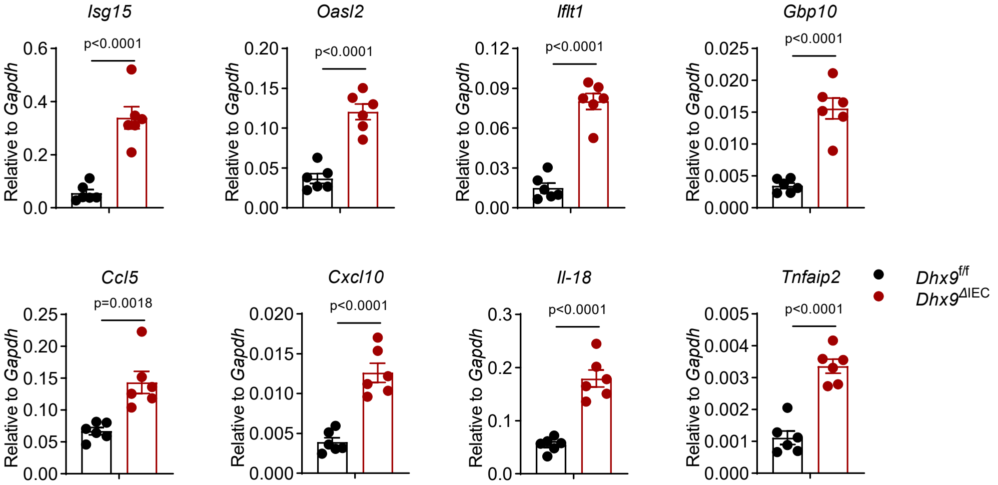

Supplementary Figure 20. DHX9 deficiency enhances cGAS-STING dependent inflammation. RT-qPCR analysis of downstream genes of cGAS-STING pathway expression in the ileum following IEC removal of *Dhx9*$^{fl//fl}$ and *Dhx9*$^{\Delta IEC}$ mice (n = 6 per group) at 8 weeks of age. All data are presented as mean ± s.e.m. Statistical analysis was performed using a two-tailed unpaired Student's *t* test. Source data are provided as a Source Data file.

Supplementary Fig. 21

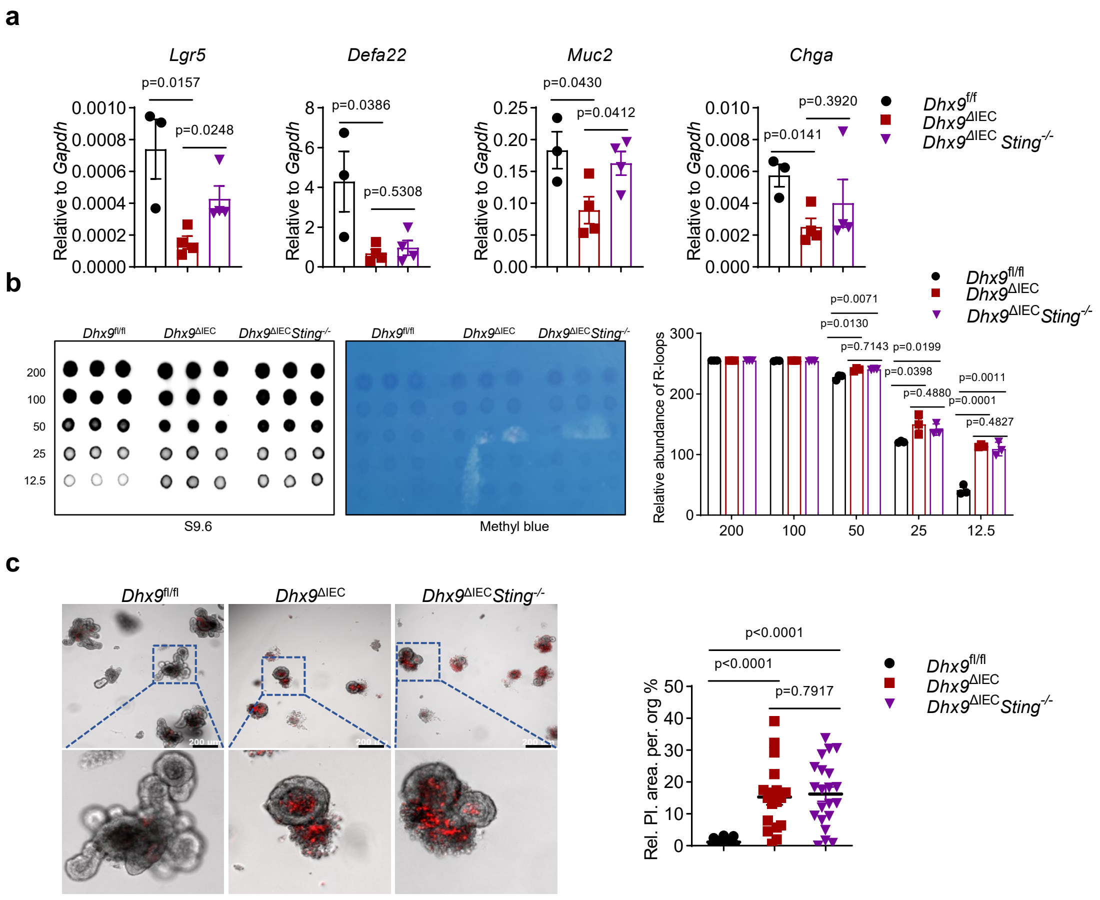

Supplementary Figure 21. IEC subpopulation dynamics and R-loop analysis in *Dhx9*$^{\Delta IEC}$ *Sting*$^{-/-}$ mice. a, RT-qPCR analysis the expression of *Lgr5*, *Defa22*, *Muc2*, and *Chga* in IECs from *Dhx9*$^{fl//fl}$ mice (n = 3), *Dhx9*$^{\Delta IEC}$ mice (n = 4), and *Dhx9*$^{\Delta IEC}$*Sting*$^{-/-}$ mice (n = 4) at 8 weeks of age. **b**, Dot blot analysis to quantify R-loops in *Dhx9*$^{\Delta IEC}$, *Dhx9*$^{fl//fl}$, and *Dhx9*$^{\Delta IEC}$*Sting*$^{-/-}$ IECs (n = 3 per group). Equal amounts of DNA were spotted onto a nitrocellulose membrane, and R-loops were detected using the S9.6 antibody. Methylene blue (MB) staining served as the loading control. Quantifications are provided on the right. All data are presented as mean ± s.e.m. **c**, PI staining of organoids derived from *Dhx9*$^{fl//fl}$, *Dhx9*$^{\Delta IEC}$, and *Dhx9*$^{\Delta IEC}$*Sting*$^{-/-}$ mice. Scale bars represent 200 µm. At least 15 organoids were counted. Three individual experiments were performed, with similar results. The panel to the right of the images provides a statistical analysis, quantifying the relative area of PI staining per organoid. Statistical analysis was performed using a two-tailed unpaired Student's *t* test. Source data are provided as a Source Data file.

Supplementary Fig. 22

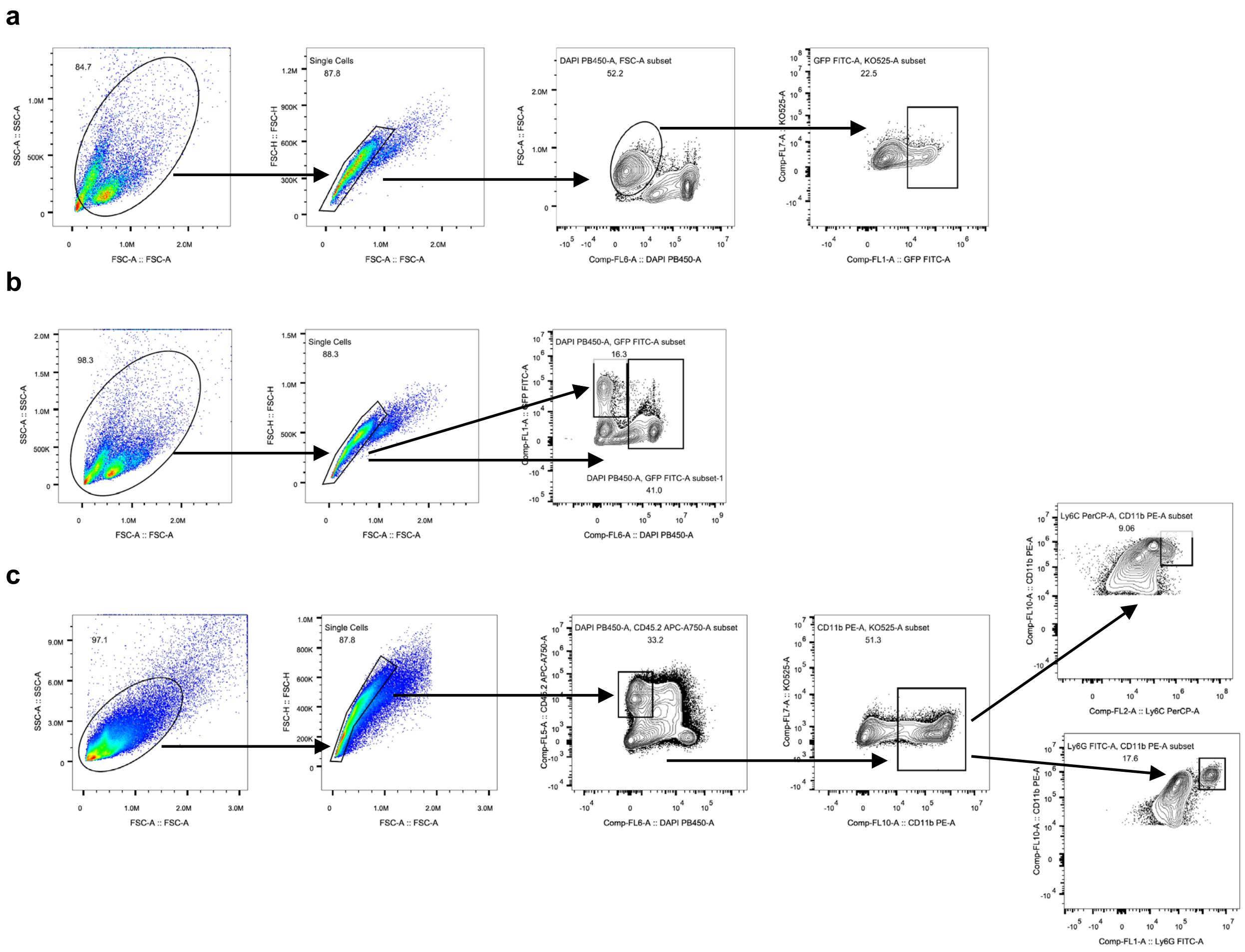

Supplementary Figure 22. Gating strategies of flow cytometric analysis. **a**, Representative flow cytometric plots showing gating strategy for GFP-positive IECs in crypt cells from corn oil-injected (control) and tamoxifen-treated *Dhx9*$^{i\Delta ISC}$ mice. **b,** Representative flow cytometric plots showing gating strategy for GFP positive lived IECs and DAPI positive IECs from *Dhx9*$^{i\Delta ISC}$ organoids after 2 days of culture treated with EtOH or 4-OH. **c**, Representative flow cytometric plots showing gating strategy for myeloid cells, including CD11b^{+}, CD11b^{+}Ly6c^{+}, and CD11b^{+}Ly6g^{+} myeloid cells in the colonic lamina propria of *Dhx9*$^{fl//fl}$ and *Dhx9*$^{\Delta IEC}$ mice on day 5 after DSS treatment.

Supplementary Table 1

Human samples information of healthy controls and IBD patients

Number	Sex	Age	Category	Usage
1	Woman	21-30	Health Control	IHC
2	Man	21-30	Health Control	IHC
3	Man	41-50	Health Control	IHC
4	Man	31-40	Health Control	IHC
5	Man	21-30	Health Control	IHC
6	Woman	21-30	Health Control	IHC
7	Woman	41-50	Health Control	IHC
8	Man	31-40	Health Control	IHC
9	Woman	51-60	Health Control	IHC
10	Woman	21-30	Health Control	IHC
11	Woman	31-40	UC Patient	IHC
12	Man	51-60	UC Patient	IHC
13	Woman	11-20	UC Patient	IHC
14	Man	61-70	UC Patient	IHC
15	Woman	31-40	UC Patient	IHC
16	Woman	31-40	UC Patient	IHC
17	Man	31-40	UC Patient	IHC
18	Woman	31-40	UC Patient	IHC
19	Man	41-50	UC Patient	IHC
20	Man	51-60	UC Patient	IHC
21	Woman	31-40	CD Patient	IHC
22	Woman	41-50	CD Patient	IHC
23	Man	21-30	CD Patient	IHC
24	Man	41-50	CD Patient	IHC
25	Woman	11-20	CD Patient	IHC
26	Man	61-70	CD Patient	IHC
27	Woman	31-40	CD Patient	IHC
28	Man	31-40	CD Patient	IHC
29	Man	11-20	CD Patient	IHC
30	Woman	31-40	CD Patient	IHC
31	man	31-40	Health Control	RT-qPCR
32	man	41-50	Health Control	RT-qPCR
33	man	41-50	Health Control	RT-qPCR
34	woman	31-40	Health Control	RT-qPCR
35	woman	51-60	Health Control	RT-qPCR
36	man	51-60	Health Control	RT-qPCR
37	woman	41-50	Health Control	RT-qPCR
38	woman	41-50	Health Control	RT-qPCR
39	man	51-60	UC Patient	RT-qPCR
40	man	71-80	UC Patient	RT-qPCR
41	man	31-40	UC Patient	RT-qPCR
42	Woman	31-40	UC Patient	RT-qPCR
43	Woman	51-60	UC Patient	RT-qPCR
44	man	41-50	UC Patient	RT-qPCR
45	Woman	21-30	UC Patient	RT-qPCR
46	Woman	41-50	UC Patient	RT-qPCR
47	Woman	21-30	UC Patient	RT-qPCR
48	man	51-60	UC Patient	RT-qPCR

Supplementary Table 2

Primers sequence for mice genotype identification in this study

Genotype primers	Sequence (5’-3’)
Dhx9$^{fl//fl}$-F	TTTTGACCAGAGTGTGCAGA
Dhx9$^{fl//fl}$-R	CCTACAATGGTTATTGTTGTAGACTGA
Villin-Cre-182/150-F	GCCTTCTCCTCTAGGCTCGT
Villin-Cre-182-R	TATAGGGCAGAGCTGGAGGA
Villin-Cre-150-R	AGGCAAATTTTGGTGTACGG
Lgr5-Cre-F	CGAGTCTGCTGTCCATTAACTC
Lgr5-Cre-R1	ACGCTTGAGGAGAGCCATTTG
Lgr5-Cre-R2	GTTCACCTAAGTGACTTGATGGC
Def-Cre-F	AGGATAACAGCATCTCCCAGTTC
Def-Cre-R	ACTTCATCAGAGGTGGCATCC
Sting-WT-F	AAACACACAGCTTTGCCGTA
Sting-KO-F	GGCAGGAAACACCAAAAAGA
Sting-Common-R	CTGTCCTCGAGCTGGTAAGG
Apc-Mut-F	TTCCACTTTGGCATAAGGC
Apc-Mut-R	TTCTGAGAAAGACAGAAGTTA
R26-wt-F	AAGGGAGCTGCAGTGGAGTA
R26-wt-R	CCGAAAATCTGTGGGAAGTC
R26-tdTomato-F	GGCATTAAAGCAGCGTATCC
R26-tdTomato-R	CTGTTCCTGTACGGCATGG

Supplementary Table 3

RT-qPCR primer sequences of mouse used in this study

RT-qPCR primers	Sequence (5'-3')
Lgr5-Forward	AGAGCCTGATACCATCTGCAAAC
Lgr5-Reverse	TGAAGGTCGTCCACACTGTTGC
Olfm4-Forward	TGAGGCCTCCAAAAGTGACC
Olfm4-Reverse	GGCCCCAGGCACCATATTTA
Ang4-Forward	GGTTGTGATTCCTCCAACTCTG
Ang4-Reverse	CTGAAGTTTTCTCCATAAGGGCT
Defa5-Forward	CTAATACTGAGGAGCAGCCAGG
Defa5-Reverse	GCAGCCTCTTATTCTACAATAGCA
Defa22-Forward	AGCAGCCAGGGGAAGAG
Defa22-Reverse	CCTCTATTGCAGCGACGT
Muc2-Forward	GTCTGCCACCTCATCATGGA
Muc2-Reverse	CAGGCAAGCTTCATAGTAGTGCTT
Chga-Forward	CAGCTCGTCCACTCTTTCCG
Chga-Reverse	CCTCTCGTCTCCTTGGAGGG
Lyz1-Forward	GGAATGGATGGCTACCGTGG
Lyz1-Reverse	CATGCCACCCATGCTCGAAT
Isg15- Forward	GGTGTCCGTGACTAACTCCAT
Isg15-Reverse	TGGAAAGGGTAAGACCGTCCT
Oasl2-Forward	TTGTGCGGAGGATCAGGTACT
Oasl2-Reverse	TGATGGTGTCGCAGTCTTTGA
Ifit1-Forward	CAAGGCAGGTTTCTGAGGAG
Ifit1-Reverse	TGAAGCAGATTCTCCATGACC
Ccl5-Forward	GCTGCTTTGCCTACCTCTCC
Ccl5-Reverse	TCGAGTGACAAACACGACTGC
Gbp10-Forward	CTGTGCAGTCTCAAACCAAG
Gbp10-Reverse	CACAAGTCGTTCTTAGG
Cxcl10-Forward	GCCGTCATTTTCTGCCTCAT
Cxcl10-Reverse	GCTTCCCTATGGCCCTCATT
Tnfaip2-Forward	AGGAGGAGTCTGCGAAGAAGA
Tnfaip2-Reverse	GGCAGTGGACCATCTAACTCG
Il-18-Forward	GACTCTTGCGTCAACTTCAAGG
Il-18-Reverse	CAGGCTGTCTTTTGTCAACGA
Gapdh-Forward	TGAGGCCGGTGCTGAGTATGTCG
Gapdh-Reverse	CCACAGTCTTCTGGGTGGCAGTG
Dhx9-Forward	GGCCAATTCCTTGCAAAGGCA
Dhx9-Reverse	CGAGGTTCAATGGGGAGTTT
