# Supplementary material for: DHX9 maintains epithelial homeostasis by restraining R-loop-mediated genomic instability in intestinal stem cells
Source: Nat Commun. 2024 Apr 9;15:3080. doi: 10.1038/s41467-024-47235-2 (PMC11004185; doi:10.1038/s41467-024-47235-2)
Supplement: Supplementary file 3 — Reporting Summary [file 41467_2024_47235_MOESM3_ESM.pdf]

Corresponding author(s): Shu Zhu, Wen Pan, Mingsong Li, Zhanju Liu

Last updated by author(s): Feb 24, 2024

Reporting Summary

Nature Portfolio wishes to improve the reproducibility of the work that we publish. This form provides structure for consistency and transparency in reporting. For further information on Nature Portfolio policies, see our Editorial Policies and the Editorial Policy Checklist.

Statistics

For all statistical analyses, confirm that the following items are present in the figure legend, table legend, main text, or Methods section.

n/a	Confirmed	
☐	☒	The exact sample size (*n*) for each experimental group/condition, given as a discrete number and unit of measurement
☐	☒	A statement on whether measurements were taken from distinct samples or whether the same sample was measured repeatedly
☐	☒	The statistical test(s) used AND whether they are one- or two-sided<br>*Only common tests should be described solely by name; describe more complex techniques in the Methods section.*
☐	☒	A description of all covariates tested
☐	☒	A description of any assumptions or corrections, such as tests of normality and adjustment for multiple comparisons
☐	☒	A full description of the statistical parameters including central tendency (e.g. means) or other basic estimates (e.g. regression coefficient) AND variation (e.g. standard deviation) or associated estimates of uncertainty (e.g. confidence intervals)
☐	☒	For null hypothesis testing, the test statistic (e.g. *F*, *t*, *r*) with confidence intervals, effect sizes, degrees of freedom and *P* value noted<br>*Give P values as exact values whenever suitable.*
☒	☐	For Bayesian analysis, information on the choice of priors and Markov chain Monte Carlo settings
☒	☐	For hierarchical and complex designs, identification of the appropriate level for tests and full reporting of outcomes
☒	☐	Estimates of effect sizes (e.g. Cohen's *d*, Pearson's *r*), indicating how they were calculated

Our web collection on statistics for biologists contains articles on many of the points above.

Software and code

Policy information about availability of computer code

Data collection

Real-time assays: CFX384 Touch Real-Time PCR Detection System (BIO-RAD)
Flow-cytometric data: CytoFlex s (Beckman Coulter), FACSverse (BD Biosciences)
HE and Fluorescent images: 3DHISTECH PannoramicMIDI II, Zeiss LSM880, THUNDER Imaging System fluorescence microscope (Leica)
Western blot data: Bio-Rad ChemiDoc MP
Transmission electron microscope data: Tecnai G2 spirit 120kV
RNAseq: Illumina HiSeq 2500
16S rRNA gene sequencing: MiSeq (Illumina)
scRNAseq: NovaSeq 6000 (Illumina)
R-loop Cut&Tag: NovaSeq 6000 (Illumina)

Data analysis	Statistical analysis: GraphPad Prism 9<br>Flow-cytometric data: FlowJo software version 10.6.2<br>Western blot data: Image Lab 5.2.1<br>HE and Fluorescent images: CaseViewer 2.4.0.119028, Image Zen 2.3 blue, ZEISS Imager, Fiji Image J<br>Real-time analysis: BioRad CFX Manager (version 3.1)<br>Raw RNA-sequencing reads were aligned to the mouse genome (mm10, GRCm38) with STAR (v2.5.3a), and gene expression quantification was performed with HTSeq (0.11.0). The resulting read counts were normalized using the DESeq2 package. Differential expression analysis was conducted using edgeR. gene ontology enrichment analyses were performed utilizing the R package clusterProfiler (v4.0.5).<br>scRNAseq: Cell Ranger and Seurat<br>R-loop Cut&Tag: Vazyme CUT_Tag_tool

For manuscripts utilizing custom algorithms or software that are central to the research but not yet described in published literature, software must be made available to editors and reviewers. We strongly encourage code deposition in a community repository (e.g. GitHub). See the Nature Portfolio guidelines for submitting code & software for further information.

Data

Policy information about availability of data

All manuscripts must include a data availability statement. This statement should provide the following information, where applicable:

- Accession codes, unique identifiers, or web links for publicly available datasets
- A description of any restrictions on data availability
- For clinical datasets or third party data, please ensure that the statement adheres to our policy

All data needed to evaluate the conclusions in the paper are present in the paper and/or the Supplementary Materials. The RNA-seq, scRNA-seq, and R-loop Cut&Tag data have been deposited in the NCBI Sequence Read Archive (SRA) BioProject (PRJNA989744, PRJNA1077865). Source data are provided with this paper.

Research involving human participants, their data, or biological material

Policy information about studies with human participants or human data. See also policy information about sex, gender (identity/presentation), and sexual orientation and race, ethnicity and racism.

Reporting on sex and gender	The specimens from patients with IBD and health control samples were obtained from First Affiliated Hospital of University of Science and Technology of China and First Affiliated Hospital of Anhui Medical University, sex and gender of specimens were presented in Supplementary table1.
Reporting on race, ethnicity, or other socially relevant groupings	the participants were recruited with no self-selection bias or other biases.
Population characteristics	Population characteristics were indicated in Supplementary table1.
Recruitment	the participants were recruited with no self-selection bias or other biases.
Ethics oversight	All subjects enrolled in this study provided written informed consent. All the human studies were approved by the Institutional Review Board for Clinical Research of First Affiliated Hospital of University of Science and Technology of China and First Affiliated Hospital of Anhui Medical University (5101116).

Note that full information on the approval of the study protocol must also be provided in the manuscript.

Field-specific reporting

Please select the one below that is the best fit for your research. If you are not sure, read the appropriate sections before making your selection.

☒ Life sciences ☐ Behavioural & social sciences ☐ Ecological, evolutionary & environmental sciences

For a reference copy of the document with all sections, see nature.com/documents/nr-reporting-summary-flat.pdf

Life sciences study design

All studies must disclose on these points even when the disclosure is negative.

Sample size	No sample-size calculations were performed. Sample size was determined to be adequate based on the magnitude and consistency of measurable differences between groups.. For in vitro and in vivo experiments, the minimum of sample size was 3 in all the cases. The sample size is stated in the figure legends.
Data exclusions	On principle, data were only excluded for failed experiments, reasons for which included suboptimal activation, misoperation and microbial contamination. No other strict data were excluded from the study.
Replication	If not stated specifically, experiments were reproduced at least three times. And all attempts at replication were successful.
Randomization	All animal- and cell-based samples in each of the group were included and no method of randomization was applied. As the results are

	qualitative, the randomization was not relevant in this study.
Blinding	Investigators were not blinded to mouse genotypes and cell lines during experiments, we need to know the genotypes of the cell lines and mouse strains. The phenotype change is beyond what can be affect by human bias, and blinding was not possible as the authors who performed the experiment also analyzed the data. The histological analyses were performed in a blinded manner.

Reporting for specific materials, systems and methods

We require information from authors about some types of materials, experimental systems and methods used in many studies. Here, indicate whether each material, system or method listed is relevant to your study. If you are not sure if a list item applies to your research, read the appropriate section before selecting a response.

Materials & experimental systems

n/a	Involved in the study
☐	☒ Antibodies
☐	☒ Eukaryotic cell lines
☒	☐ Palaeontology and archaeology
☐	☒ Animals and other organisms
☒	☐ Clinical data
☒	☐ Dual use research of concern
☒	☐ Plants

Methods

n/a	Involved in the study
☒	☐ ChIP-seq
☐	☒ Flow cytometry
☒	☐ MRI-based neuroimaging

Antibodies

Antibodies used	Western blotting: Anti-DHX9 (ab26271, Abcam, 1:2000), anti-γH2AX (GB111841, Servicebio, 1:200), anti-GAPDH (60004-1-Ig, Proteintech, 1:2000), anti-ACTIN (81115-1-RR, Proteintech, 1:2000).<br>Immunofluorescent staining: Dilution of each antibody is 1:200. anti-Ki67 (GB111141, Servicebio), anti-Lysozyme (GB11345, Servicebio), anti-MUC2 (GB11344, Servicebio), anti-E-cadherin (GB12082, Servicebio), anti-Nucleolin (10556-1-AP, Proteintech), anti-Cleved-Caspase-3 (9661, Cell Signaling Technology), DHX9 (67153, Proteintech).<br>Dot-Blot: anti-S9.6 (ENH001, Kerafast, 1:200)<br>FACS analysis: Dilution of each antibody is 1:100. APC/Cy7 anti-CD45.2 (Biolegend, clone 104), PE anti-Ep-CAM (Biolegend, clone G8.8), PE anti-CD11b (Biolegend, clone M1/70), FITC anti-Ly6G (Biolegend, clone 1A8), PerCP/Cy5.5 anti-Ly6C (Biolegend, clone HK1.4).
Validation	All antibodies are commercially available and have been verified by the manufacturers according to the immunoblots and/or images on their websites.<br>anti-DHX9: https://www.abcam.com/products/primary-antibodies/rna-helicase-a-antibody-ab26271.html<br>anti-γH2AX: https://www.servicebio.cn/goodsdetail?id=4702<br>anti-GAPDH: https://www.ptglab.com/products/GAPDH-Antibody-60004-1-Ig.htm<br>anti-ACTIN: https://www.ptglab.com/products/beta-actin-Antibody-81115-1-RR.htm<br>anti-Ki67: https://www.servicebio.cn/goodsdetail?id=2828<br>anti-Lysozyme: https://www.servicebio.cn/goodsdetail?id=1174<br>anti-MUC2: https://www.servicebio.cn/goodsdetail?id=1173<br>anti-E-cadherin: https://www.servicebio.cn/goodsdetail?id=597<br>anti-S9.6: https://www.kerafast.com/productgroup/432/anti-dna-rna-hybrid-s96-antibody<br>anti-Nucleolin: https://www.ptgcn.com/products/NCL-Antibody-10556-1-AP.htm<br>anti-Cleved-Caspase-3: https://www.cellsignal.cn/products/primary-antibodies/cleaved-caspase-3-asp175-antibody/9661<br>anti-DHX9: https://www.ptgcn.com/products/DHX9-Antibody-67153-1-Ig.htm<br>APC/Cy7 anti-CD45.2: https://www.biolegend.com/en-us/products/apc-cyanine7-anti-mouse-cd45-2-antibody-3906<br>PE anti-Ep-CAM: https://www.biolegend.com/en-us/products/pe-anti-mouse-cd326-ep-cam-antibody-4726<br>PE anti-CD11b: https://www.biolegend.com/en-us/products/pe-anti-mouse-human-cd11b-antibody-349<br>FITC anti-Ly6G: https://www.biolegend.com/en-us/products/fitc-anti-mouse-ly-6g-antibody-4775<br>PerCP/Cy5.5 anti-Ly6C: https://www.biolegend.com/en-us/products/percp-cyanine5-5-anti-mouse-ly-6c-antibody-5967

Eukaryotic cell lines

Policy information about cell lines and Sex and Gender in Research

Cell line source(s)	HeLa cells were purchased from ATCC.
Authentication	All cell lines were authenticated on the basis of their morphology and growth condition.
Mycoplasma contamination	The cells were not tested for mycoplasma contamination.
Commonly misidentified lines (See ICLAC register)	No commonly misidentified cell lines were used.

Animals and other research organisms

Policy information about studies involving animals; ARRIVE guidelines recommended for reporting animal research, and Sex and Gender in Research

Laboratory animals	All mice were housed in cages with five mice per cage and kept on in a regular 12h light/12h dark cycle (lights on at 7:00 am). The temperature was 24±2 degree Celsius and humidity was 40-70%, with a regular chow diet. 6–12-week-old and sex-matched mice in C57BL/6 background were used in all assays. All animal experiments were approved by the Ethics Committee of the University of Science and Technology of China.Villin-Cre and Lgr5-EGFP-IRES-CreERT2 mice were obtained from the Jackson Laboratory. The Defa6-Cre mice were kindly provided by Richard Blumberg (Harvard Medical School, Boston). The Sting−/− mice were kindly provided by Daxing Gao (University of Science and Technology of China, Hefei). Apcmin/+ mice, and Ai14 reporter mice (Rosa26lsl-tdTomato) were provided by Richard A. Flavell from Yale University. Dhx9fl/fl mice were crossed with Villin-Cre mice, Lgr5-EGFP-IRES-creERT2 mice, or Defa6-Cre mice to generate Dhx9 depletion in IECs (Dhx9ΔIEC), Paneth cells (Dhx9ΔPaneth) and Lgr5+ stem cells (Dhx9iΔISC) respectively. Lgr5-EGFP-IRES-creERT2 mice were crossed them with Rosa26lsl-tdTomato to generate Lgr5-EGFP-CreERT2: Rosa26lsl-tdTomato mice. Dhx9fl//fl Villin-Cre mice (Dhx9ΔIEC) were crossed with Sting−/− mice to generate double-knockout mice. . Sample sizes for mouse experiments were empirically determined, and mice were randomly assigned to control or experimental group. For all experiments, littermates were used as control indicated in each figure.
Wild animals	The study did not involve wild animals.
Reporting on sex	Both female and male mice were included in this study.
Field-collected samples	The study did not involve samples collected from the field.
Ethics oversight	All animal studies were approved by the institutional review board at University of Science and Technology of China (2021-N(A)-271).

Note that full information on the approval of the study protocol must also be provided in the manuscript.

Flow Cytometry

Plots

Confirm that:

- [x] The axis labels state the marker and fluorochrome used (e.g. CD4-FITC).
- [x] The axis scales are clearly visible. Include numbers along axes only for bottom left plot of group (a 'group' is an analysis of identical markers).
- [x] All plots are contour plots with outliers or pseudocolor plots.
- [x] A numerical value for number of cells or percentage (with statistics) is provided.

Methodology

Sample preparation	The information was included in the Methods section.
Instrument	CytoFlex s (Beckman Coulter).
Software	FlowJo software version 10.6.2.
Cell population abundance	Sort-purification was carried out using BD FACSAria III, with >98% purity.
Gating strategy	The gating strategy will be presented in Extended data.

- [x] Tick this box to confirm that a figure exemplifying the gating strategy is provided in the Supplementary Information.
